# 3D free-assembly modular microfluidics inspired by movable type printing

**DOI:** 10.1038/s41378-023-00585-1

**Published:** 2023-09-11

**Authors:** Shaoqi Huang, Jiandong Wu, Lulu Zheng, Yan Long, Junyi Chen, Jianlang Li, Bo Dai, Francis Lin, Songlin Zhuang, Dawei Zhang

**Affiliations:** 1 Engineering Research Center of Optical Instrument and System, the Ministry of Education, Shanghai Key Laboratory of Modern Optical System, University of Shanghai for Science and Technology, Shanghai, 200093 China; 2 Institute of Biomedical and Health Engineering, Shenzhen Institute of Advanced Technology, Chinese Academy of Sciences, Shenzhen, 518055 China; 3 Department of Physics and Astronomy, University of Manitoba, Winnipeg, MB R3T 2N2 Canada

**Keywords:** Microfluidics, Engineering

## Abstract

Reconfigurable modular microfluidics presents an opportunity for flexibly constructing prototypes of advanced microfluidic systems. Nevertheless, the strategy of directly integrating modules cannot easily fulfill the requirements of common applications, e.g., the incorporation of materials with biochemical compatibility and optical transparency and the execution of small batch production of disposable chips for laboratory trials and initial tests. Here, we propose a manufacturing scheme inspired by the movable type printing technique to realize 3D free-assembly modular microfluidics. Double-layer 3D microfluidic structures can be produced by replicating the assembled molds. A library of modularized molds is presented for flow control, droplet generation and manipulation and cell trapping and coculture. In addition, a variety of modularized attachments, including valves, light sources and microscopic cameras, have been developed with the capability to be mounted onto chips on demand. Microfluidic systems, including those for concentration gradient generation, droplet-based microfluidics, cell trapping and drug screening, are demonstrated. This scheme enables rapid prototyping of microfluidic systems and construction of on-chip research platforms, with the intent of achieving high efficiency of proof-of-concept tests and small batch manufacturing.

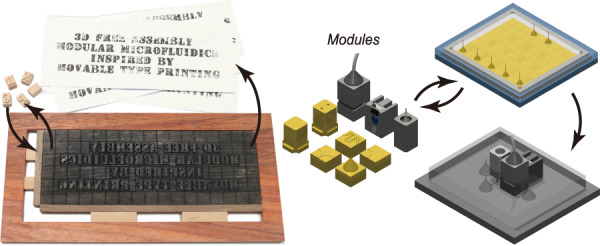

## Introduction

In the past three decades, microfluidic technology has significantly progressed toward broad applications in scientific and engineering fields, including biological research, biomedical diagnosis, material synthesis and analytical chemistry^1–3^. The rapid growth of microfluidic technology benefits from the development of advanced microfabrication techniques, such as soft lithography, laser direct writing and 3D printing techniques^4–7^. Various microfluidic microstructures have been developed to realize laboratory processes^8–13^. In general, microstructures are all designed and integrated together on a single chip to achieve the overarching goal of lab-on-a-chip (LOC). The fabrication of microfluidic chips with a monolithic structure is suitable for mass production, but may not be a favorable strategy in early stages of research and development because partially updating a structure is not flexible. In addition, modularization of microfluidic structures is an alternative strategy to construct microfluidic systems with pieces of modules, allowing reconfigurability, flexibility and variety in use. Due to its outstanding features, modular microfluidics has drawn great attention in the microfluidic community^14–19^.

Nearly universally, those modular microfluidic systems reported previously are realized by assembling a group of modules together. Each module possesses a particular structure corresponding to a specific microfluidic function. The modules can be produced in various techniques. One of the popular technique to produce microfluidic modules is based on the soft lithography^20^. Therein, dozens of microstructures are fabricated on wafers and then transferred to an elastomeric material, e.g., polydimethylsiloxane (PDMS), thus forming modules. These modules can be flexibly assembled into a whole system by adhesion^21,22^. Moreover, microfluidic modules can be directly produced by 3D printing^23–25^. However, it is a great challenge for 3D printing techniques to simultaneously fulfill the requirements of high printing resolution, high surface smoothness and utilization of materials with biochemical compatibility and high optical transparency. Using PDMS modules cast from 3D printed molds or aluminum molds provides a compromise solution^26–28^. However, it is inefficient to prepare piecewise disposable PDMS modules, especially for small batch production. Modification of standardized injection-molded units, e.g., LEGO^®^ bricks, is a straightforward way to produce customized modules, but it is challenged to fabricate 3D structures on the scale of tens of micrometers^29^.

**Fig. 1:**
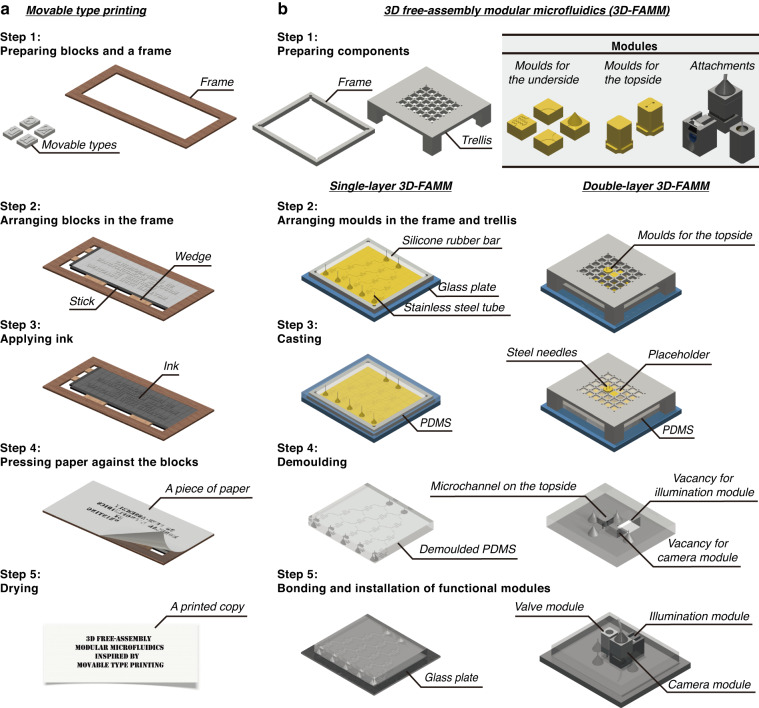
Schematic presentation of movable type printing and 3D free-assembly modular microfluidics. **a** Illustration of the main steps of movable type printing. **b** Fabrication procedure of 3D free-assembly modular microfluidics

Woodblock printing technology is a significant invention of China’s Tang Dynasty (AD 606–906), which enabled the dissemination of knowledge and the spread of culture. Woodblock printing is somewhat analogous to soft lithography in microfluidic fabrication. For example, in woodblock printing, multiple copies of scripts could be easily produced by brushing paper over an inked wooden board on which text or images were engraved. However, carving hundreds of wooden boards for different scripts was time-consuming, and correction of mistakes is difficult. Around AD 1040, movable type printing technology was invented during the Northern Song Dynasty of China. Printing typefaces with elements, e.g., alphabetic characters, figures or symbols, were prepared by engraving or casting (Fig. 1a, Supplementary Fig. 1a). In typesetting, the types were assembled to form pages and inked for copying. In contrast to woodblock printing, movable type printing has significantly high efficiency for producing different scripts. Inspired by movable type printing, we developed a 3D free-assembly modular microfluidics (3D-FAMM) scheme for microfluidic fabrication. Molds for fluid control and functional attachments, e.g., valve, illumination source and microscopic camera, were modularized into standardized parts. A microfluidic system was formed by replicating assembled molds and installing attachments. The extraordinary features of 3D-FAMM are as follows: (1) flexibility in the construction of all-in-one microfluidic systems; (2) reusability of modular molds and attachments; (3) wide selection of materials with biochemical compatibility and optical transparency; (4) standardization of size and interconnection and standard operating procedure (SOP); (5) ease of fabrication; and (6) high cost efficiency. Using this platform, various 3D-FAMM systems were demonstrated for several applications, including complex concentration gradient generation, droplet generation and manipulation, cell trapping and cell coculture.

## Results

### Concept

The 3D-FAMM is inspired by movable type printing technology. The fabrication procedure of 3D-FAMM, referring to the main steps of movable type printing (Fig. 1a), is illustrated in Fig. 1b. The modules are divided into two groups, i.e., mold and attachment. Instead of producing a mold for an entire microfluidic structure, pieces of molds with small portions of structures, e.g., microchannel, splitter and mixer, are 3D printed and could be arranged into any desired large structure. In addition to the molds, a variety of attachments, including light sources, microscopic camera and pneumatic valves, are also prepared, which could be installed onto the microfluidic chips to enrich functionality. The design of all the molds and the attachments follows a standard that defines the size and the connection.

The entire microfluidic structure is created by arranging the molds in a frame and a trellis. On the first layer, the molds are assembled in the frame. Silicone rubber bars are inserted into the frame to compact the molds (Supplementary Fig. 2a). Stainless steel tubes are inserted into the molds on demand, for example, inlets, outlets and interlayer connections. To create the microstructure on the second layer, the trellis is placed on the frame, and the molds for the topside are mounted into the trellis (Supplementary Fig. 2b). Once the molds are all assembled, PDMS is poured into the frame. After curing for 6 h at 60 °C, the PDMS was demolded from the molds. The underside of the PDMS is bonded to a glass plate, and the topside is covered with PDMS films. The vacancies in the microfluidic chip are reserved for the modular attachments. The fabrication process is further depicted in Supplementary Movie 1 and Supplementary Movie 2.

The 3D-FAMM kit, including the molds, attachments, frame and trellis, is shown in Supplementary Fig. 1b. The representative molds and attachments are listed in Supplementary Table 1. Each mold or attachment is used as a module. The modular size is defined as 5 × 5 mm^2^ (length × width). The height of the molds for the underside is 3 mm. The molds for both sides are aligned. The molds are produced by projection microstereolithography 3D printing. Feature sizes up to 15 μm and thin thickness layers of 5 μm can be achieved^30,31^.

### Characterization

Consistency in the dimensions of the molds affects the assembly of the microstructures and the alignment of the microchannels. The fabrication errors in length/width and height were −4.9 to 4.4 μm and –1.6 to 1.3 μm, respectively (Supplementary Fig. 3a). The roughness of the mold (the average of surface heights *Ra* < 9 nm) can be neglected (Supplementary Fig. 4). In our design, the microchannel interface is standardized as 130 μm × 100 μm (width × height), which is tolerable to the worst-case cumulative error of 20 molds in series (details provided in Supplementary Fig. 5). Furthermore, two silicone rubber bars with a width of 3 mm are inserted into the frame to compress the molds. The deformation, i.e., the strain *ε* < 0.004, is in the linear elastic region (the analysis of the mold deformation can be found in Supplementary Note 1). Once the compression applied on the molds is withdrawn, the molds can be recovered into their original shape. Thus, the molds would not be damaged and could be reused in different applications.

**Fig. 2:**
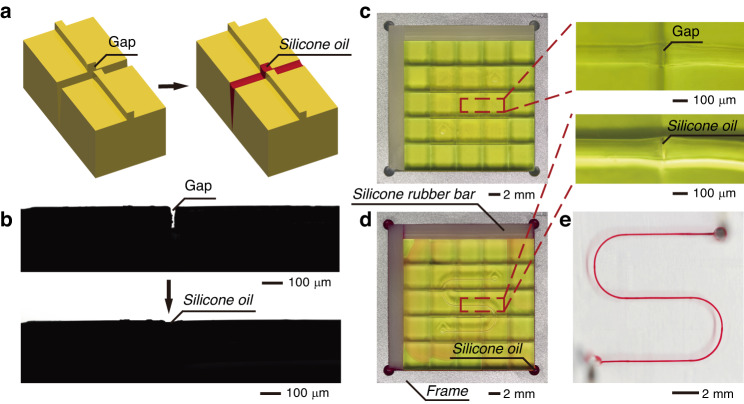
Assembly of the 3D-FAMM chips. **a** The filling of the gap between the molds. **b** Microscope images of the side view of the two assembled molds before and after filling the gap. **c** The molds assembled for an S-shaped microchannel. Gaps could be observed between the molds. **d** The assembled molds in the frame where silicone oil was added in the four corners. Silicone oil filled the gaps. **e** Image of the 3D-FAMM chip with an S-shaped microchannel. Red ink was injected into the microchannel

3D printing can hardly realize perfect end faces of the connecting microchannels. The gaps caused by the rough end faces form partition walls in the replicated microchannels, resulting in flow obstruction. These gaps were filled with silicone oil (Fig. 2a, b). Fig. 2c shows the molds assembled in the frame to establish an S-shaped microstructure. The width of the microchannel is 100 μm in the center of the mold and gradually widened to 130 μm at the edge to conform to the interface standard. The height of the microchannel is 100 μm. After assembling the molds, silicone oil was dripped into the holes on the four corners of the frame. In the experiment, the silicone oil was dyed with red solvent for tracing. The silicone oil spread over the structure through the gaps due to the capillary force (Fig. 2d). Because the density of the silicone oil (*ρ*_*Oil*_ = 1.208 g/cm^3^) is higher than that of PDMS (*ρ*_*PDMS*_ = 1.03 g/cm^3^), the silicone oil remained in the gaps during replication of the structure. Fig. 2e demonstrates the injection of the red ink into the finished chip. The fluid flowed smoothly along the S-shaped microchannel. Neither leakage nor blockage was observed, proving the feasibility of the 3D-FAMM.

**Fig. 3:**
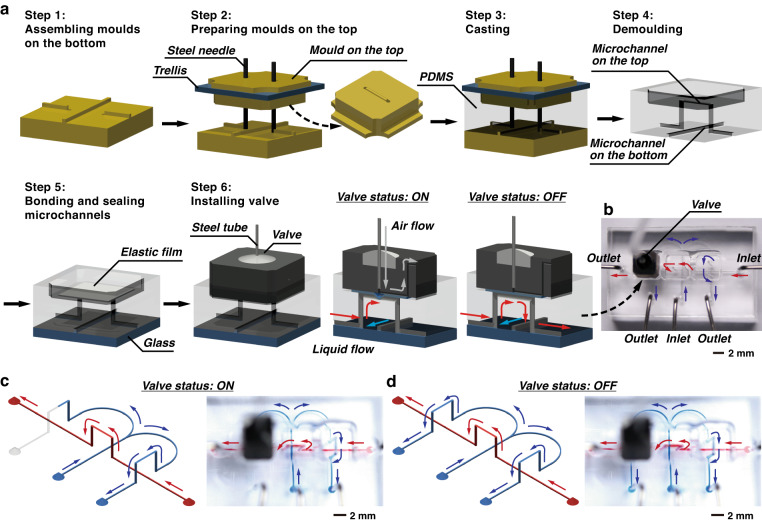
Fabrication of the 3D-FAMM chips. **a** Fabrication of a double-layer 3D-FAMM chip consisting of a straight microchannel and an m-shaped microchannel crossing each other. A valve module was equipped at a crossing point. **b** The fully assembled double-layer 3D-FAMM chip. **c**, **d** Demonstration of the flows in the double-layer 3D-FAMM chip. Red ink was injected into the straight microchannel, and blue ink was injected into the m-shaped microchannel. The valve embedded at one of the branches was turned on (**c**) to block the blue flow, and then the valve was turned off (**d**) to allow the blue flow to pass through

Fig. 3a demonstrates the fabrication and operation of a double-layer 3D-FAMM. The molds for the undersides were assembled in the frame. Then, the trellis was placed on the frame. Molds for the topside were mounted into the trellis, aligning to the molds on the bottom. Steel needles were inserted in the molds and connected the microstructures on the two layers. After demolding, the underside of the PDMS chip was bonded to a glass plate. On the topside of the chip, microchannels were created on the bottom of the pits. PDMS films with a thickness of 20 μm were bonded to the bottom of the pits to seal the microchannels.

Pneumatic valves are used to control the fluid motion. Each valve was embedded into the pit and actuated by air pressure. When a valve was turned on, pneumatic pressure was applied to the neighboring elastic film, and the film was deformed to block the channel. The film exhibited elastic recovery after the pneumatic pressure was stopped, such that the channel became unobstructed.

Fig. 3b illustrates the fully assembled 3D-FAMM chip. The chip includes two microchannels, i.e., an m-shaped microchannel and a straight microchannel, which intersect each other. At each crossing point, vertical interconnect accesses (VIAs) connect the channel on the first layer and a bypass on the second layer. A pneumatic valve was placed on one of the crossing points. Blue ink was injected into the m-shaped microchannel, while red ink was injected into the straight microchannel. The flow rates of the two fluids were 1 μL/min. The valve was turned on at first to block one branch of the m-shaped microchannel (Fig. 3c). After splitting into the two branches, the blue ink stopped at the valve in one branch, and in the other branch, the ink strode over the straight microchannel. The red ink flowed along the straight channel and crossed the bypass on the second layer. Once the valve was turned off, the blue ink flowed out of the chip from the two outlets (Fig. 3d). The red ink kept flowing in the other separate microchannel. No mixing or leakage of the fluids occurred.

In the 3D-FAMM, complex microfluidic structures can be flexibly arranged using the two layers. The interlayer VIAs and the bypasses establish the linkages of the microstructures, counteracting geometric constraints. In addition, active fluid-motion control could achieve architectures for reconfigurable and programmable microfluidics.

### Concentration gradient generation

Engineering a concentration gradient is an essential technique in various biological and chemical applications, including drug screening, immunoassays and material synthesis^32–35^. The creation of microenvironments with varying concentrations in microfluidics has attracted great research attention. Many concentration gradient generation schemes have been demonstrated^36–38^. Christmas-tree design is a popular scheme that is widely used in many applications^39–41^, where two or more fluids with different concentrations are injected into the network with the pattern in Pascal’s triangle. After stage-by-stage fluid splitting and mixing, a series of fluids with a concentration gradient can be generated. Complex concentration profiles can be generated by designing mixing channels with specific hydraulic resistances.

**Fig. 4:**
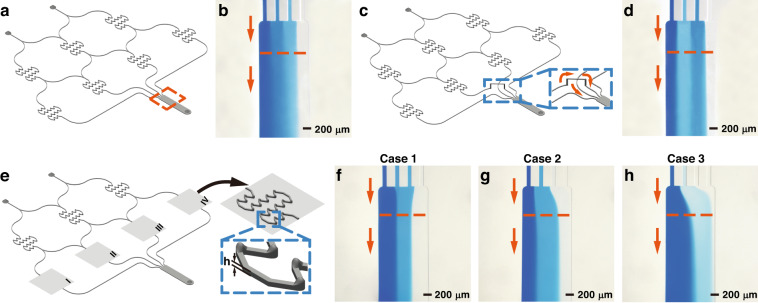
Generation of a concentration gradient with complex profiles. **a** A conventional Christmas tree design realized by 3D-FAMM. All the mixers have identical dimensions. **b** Image of the flow with a stepwise concentration profile captured in the microfluidic chamber as marked with an orange box in **a**. **c** A Christmas tree design with the two outputs at Stage 2 crossing each other. **d** Image of the flow with a concentration profile for the structure shown in **c**. **e** A Christmas tree design using mixers of different dimensions. **f**–**h** Images of the flow with different concentration profiles

The use of our 3D-FAMM for Christmas-tree concentration gradient generation is demonstrated (Fig. 4). A 9 × 10 mold array, including inlet/outlet, 90°-turn channel, 1-to-2 splitter, mixer and chamber molds, was assembled to establish a two-stage Christmas tree structure (Supplementary Fig. 6). The mixer has a zigzag structure (Supplementary Fig. 7). At every turn of the microchannel, there is a slight bulge. These bulges contributes to the enhancement of the advection. Thus, the mixing efficiency is significantly improved over a zigzag structure without bulges (Supplementary Fig. 8).

In the experiment, blue ink and deionized water were separately injected into the chips from the two inlets. The flow rate of the injection was 1 μL/min. The change in the fluid concentration, which was linearly proportional to the color change, was observed in the chamber and derived by colorimetric analysis (see Supplementary Note 2 and Supplementary Fig. 9). When the mixers are identical, the hydraulic resistances in the branching channels are the same, and the splitting ratio is 1:1 (Fig. 4a). In the chamber, the concentration of the fluid presents a stepwise growth with four levels equally distributed in the transverse direction along the flow (Fig. 4b, Supplementary Fig. 10a). Moreover, the outputs of Stage 2 can be flexibly exchanged by simply modifying the structure. The 4-channel fan-in/fan-out mold is changed to the one with a crossover, as highlighted in the blue dashed box in Fig. 4c. A bypass is established on the second layer to connect the two ends of the microchannels on the first layer. Because the middle two outputs of Stage 2 are exchanged spatially, the merged fluid demonstrates a stepwise concentration profile with fluctuations (Fig. 4d, Supplementary Fig. 10b). This concentration profile is not monotonous and cannot be generated in the conventional single-layer Christmas-tree structure.

Moreover, with the change in the hydraulic resistance in the network, the concentration profile can be adjusted. The hydraulic resistance of the mixer is determined by its dimension. To maintain the footprint of the mixer within the modular size, mixers with different heights were fabricated (Fig. 4e). The three mixers in Stage 1 were maintained constant (height = 100 μm), while the four mixers in Stage 2 were replaced with those listed in Supplementary Fig. 10c. The inequality of the hydraulic resistances after splitting results in imbalanced flow rates in the two branching channels. The concentration profiles using different mixers are measured (Fig. 4f, h, Supplementary Fig. 10d). The arrangement of the hydraulic resistances in the network has a significant influence on the flow rate and the concentration at the output. Consequently, the concentration gradients in the merged flows in the three cases are quite different. The 3D-FAMM Christmas-tree concentration gradient generator offers the potential to realize desirable concentration profiles by arranging mixers with appropriate hydraulic resistance. The 3D-FAMM exhibits a superior capability of modifying a part of the structure in an easy way, which is favorable in the laboratory trials and production testing stages.

### Droplet generation and manipulation

**Fig. 5:**
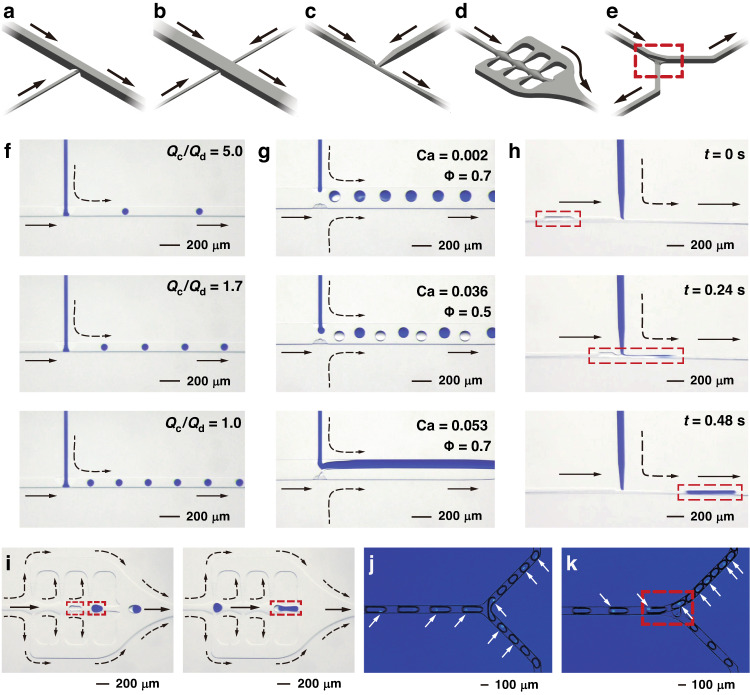
Droplet generation and manipulation. **a**–**e** The design of the structures for droplet generation (**a**, **b**), droplet injection (**c**), droplet merging (**d**) and droplet splitting (**e**). **f** The generation of droplets at different ratios of the injection rates. **g** The generation of merging droplets, alternating droplets and laminar flow. **h** The montage of droplet injection. **i** The montage of droplet merging. **j** Droplet splitting in a symmetric Y-junction structure. **k** Droplet splitting in a Y-junction structure with a 3D obstacle in one of the branches

Droplet-based microfluidics or digital microfluidics provide a high-throughput, low-volume reaction platform that can be leveraged to conduct assays with extremely high efficiency using limited specimens^42–45^. The key functions of droplet-based microfluidics are demonstrated by the 3D-FAMM system. The 3D-FAMM design for droplet generation and manipulation is shown in Fig. 5a-e (Supplementary Fig. 11). These modules can be employed to construct various droplet-based microfluidic systems.

The droplet generator has a modified T-junction structure with a 3D nozzle configuration (see the mechanism of droplet generation in Supplementary Note 3). Compared to a generator with a conventional T-junction, the generator with the 3D structure can generate smaller droplets at a higher rate (Supplementary Fig. 12). Because the cross section shrinks during operation of the 3D structure, it takes less time for the tongue of the discrete phase to fill in the main channel. In addition, the large curvature of the neck through the nozzle allows the continuous phase to easily squeeze and break the tongue. Fig. 5f shows the formation of the droplets (Supplementary Movie 3). When the flow rate of the discrete phase is fixed, the generation rate can be increased, and the size of the droplets can be reduced with increasing flow rate of the continuous phase, as shown in Supplementary Fig. 13a. If the two T-junction structures are arranged on the two sides of the main channel (Fig. 5b), then generation of alternating droplets, merging droplets and laminar flow can be achieved with different capillary numbers (*Ca*) and flow rate fractions (*Φ*) (Fig. 5g, Supplementary Fig. 13b and Supplementary Movie 3).

Various modules for droplet manipulation can be added after the droplet generator. For example, an injection module can be employed to add fluid into the droplets (Fig. 5c). In this design, the main channel suddenly narrows before the injection point, forming a Venturi tube. The abrupt change in the channel results in a pressure drop at the injection section, as calculated in Supplementary Fig. 14. Thus, the Venturi effect allows the injection to be operated at a low pressure, ensuring its stability. Moreover, the droplets are elongated in the narrow channel so that large-volume injection can be realized, as shown in Fig. 5h and Supplementary Movie 3. The injection volume can be flexibly adjusted by controlling the injection rate (Supplementary Fig. 15). A wide range of injection volumes from 0.06 nL to 0.44 nL can be achieved.

A module with a fishbone structure is designed for droplet merging (Fig. 5d). The main fishbone channel has three fusiform chambers, which connect to the two auxiliary channels on the two sides. Since the distributary fluid flows away from the main channel into the auxiliary channels, the flow rate in the latter chamber is lower than that in the former chamber (Supplementary Fig. 16). Droplet merging was demonstrated using alternating droplets, as shown in Fig. 5i and Supplementary Movie 3. When a droplet flowed along the main channel, it slowed down in every chamber as if it was waiting for the next droplet. Once the subsequent droplet hit the previous droplet, the two droplets merged.

Furthermore, droplet splitting is demonstrated in a Y-shaped-structure module (Fig. 5j, k and Supplementary Movie 3). In this droplet generation, HBEC3-KT cells stained with 4’,6-diamidino-2-phenylindole (DAPI) were encapsulated into the droplets. Once a droplet passed through the Y-junction, it split into two daughter droplets. The daughter droplets carrying the cells randomly entered the two branches when they had an identical structure (Fig. 5j). If a 3D obstacle was included in one of the branches, i.e., a sudden change in height from 100 μm to 10 μm, the daughter droplets carrying the cells only entered the other branch (Fig. 5k).

### Cell trapping and co-culture

Although the printing resolution (line width ≥ 15 μm) of the 3D printing hinders the design of micrometer-scale structures, the layer thickness (5 μm) is comparable to the size of a single cell. Therefore, modules for cell trapping and co-culture have been developed.

**Fig. 6:**
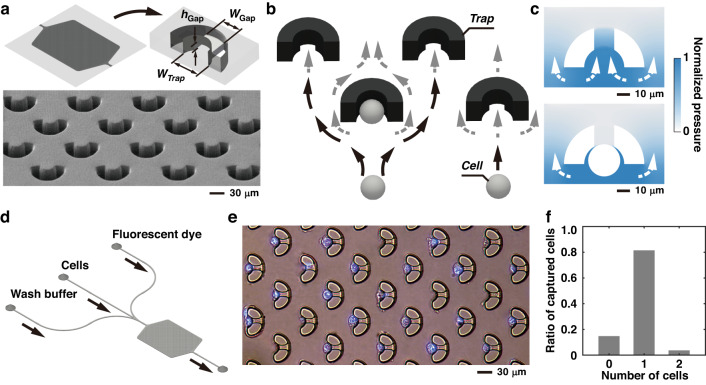
Cell trapping and fluorescence staining. **a** Design and scanning electron microscopic (SEM) image of the cell-trapping mold. **b** Schematic diagram of the demolded traps and the operation principle of microparticle trapping. **c** Simulation of the particle trapping when the trap is vacant or occupied. **d** The platform for cell trapping and fluorescence staining. **e** Microscopic image of the cells captured by the trap array and stained with DAPI fluorescent dye. **f** Trapping efficiency indicates that most traps (>81%) capture single cell

Fig. 6a illustrates the cell trapping mold in which there are up to 6000 traps (Supplementary Fig. 17). After demolding, each PDMS trap has a U-shaped structure with an entrance of 30 μm width. A small gap with a width, *w*_*Gap*_, of 15 μm and a height, *h*_*Gap*_, of 5 μm is on the bottom of the trap. Due to the existence of the gaps, the pressure drop across the vacant traps drives the microparticles to enter the traps along the streamlines, as depicted in Fig. 6b, c and Supplementary Fig. 18. When the traps are occupied, the particles bypass the traps and are captured by the succedent traps. A microfluidic platform for cell trapping and fluorescence staining is built using the cell-trapping module (Fig. 6d). HBEC3-KT cells, DAPI fluorescent dye and wash buffer were successively injected into the microfluidic chip. The cells could be efficiently captured by the trap array (Fig. 6e), indicating that the microstructure with high manufacturing precision is capable of intercepting microscale elastic particles. Since the volume of the traps matched the size of the cells, the single-cell trapping efficiency reached 81.5% (Fig. 6f).

**Fig. 7:**
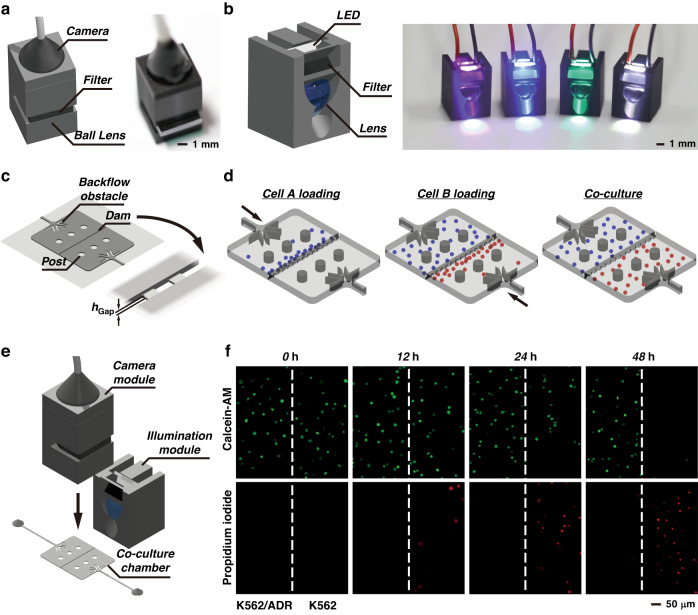
Cell coculture for drug-resistance analysis. **a** Design and photograph of the camera module. **b** Design and photograph of the illumination module. **c** Design of cell coculture mold. **d** Illustration of cell loading and coculture. **e** Schematic diagram of the cell coculture platform including microscopic imaging components. **f** Fluorescence images of K562/ADR and K562 cells cultured in the Adriamycin

In the cell co-culture demonstration, camara and illumination modules are developed for microscopic imaging, as shown in Fig. 7a, b and Supplementary Fig. 19. In the camera module, there is an endoscope camera, a ball lens and an optical bandpass filter (center wavelength at 450 nm, 520 nm or 620 nm). The field of view is 1300 × 1300 μm, and the resolution is 2.2 μm (Supplementary Fig. 20). The illumination module is equipped with an LED, an optical bandpass filter and a hemispherical lens. A white-light spot or a light spot with a specific wavelength (365 nm, 480 nm or 535 nm) is generated next to the module. These attachments provide a promising solution to establish all-in-one microfluidic platforms. In addition, a cell co-culture module with two chambers was demonstrated (Fig. 7c). Two groups of cells can be injected into the two chambers separately and isolated by a dam that has small gaps allowing interflow between the chambers, as depicted in Fig. 7d. The cell co-culture system was applied to drug screening (Fig. 7e). K562 and K562/ADR cells were loaded into the two chambers. Adriamycin (500 ng mL^−1^) was added to the chambers. In the cell viability assay, calcein acetoxymethyl ester (calcein-AM) and propidium iodide (PI) fluorescent dyes and wash buffer were successively injected into the chips. Then, the illumination modules and the camera module were mounted onto the chip to excite the cells and record the fluorescence emission, as shown in Fig. 7f. The consistent detection of Calcein-AM green fluorescence emission in K562/ADR cells over 48 h confirms the characteristics of drug resistance, while the change in fluorescence emission in K562 cells indicates that the cells deteriorate gradually and are sensitive to the drug.

## Discussion

In summary, we have presented a novel scheme, 3D-FAMM, for the fabrication and modification of diverse microfluidic systems. The scheme is inspired by movable type printing technology. Our standardized modules with 3D structures can be flexibly arranged in the frame and trellis architecture to construct an entire mold for the double-layer microfluidic structure. The standard platform regulates the dimension of the modules, the structure of the inter/intralayer connection and the assembling procedure. The misalignment of the microchannels is tolerable with at least 20 modules connected in series. The deformation of the modules inserted in the frame occurs in the linear elastic region. Thus, the modules are not damaged and can be reused in the other systems. In addition, any gaps between the modules resulting from tolerance of the 3D printing process can be filled with silicone oil. As a result, the microchannels in the replicated microfluidic chips are unobstructed. Moreover, the double-layer feature would benefit microfluidic systems with complex networks.

A wide variety of modules have been developed for flow control, droplet operation, cell interception and isolation, pneumatic valve control, illumination and imaging. In the current 3D-FAMM scheme, the projection micro-stereolithography 3D printing technique is adopted for fabricating modules. Alternatively, it is feasible to apply other advanced 3D manufacturing techniques, e.g., direct laser writing and electrodeposition-based 3D printing^46^, to fabricate modules. Furthermore, the library of the modules could be expanded to include more molds for fluid control and functional attachments that replace the peripheral bulky equipment.

The 3D-FAMM scheme provides a novel technique for prototyping microfluidic systems. During research and development of tools used in the abovementioned applications, including concentration gradient generation, droplet generation and manipulation, cell trapping and drug screening, it is convenient to construct and modify microfluidic systems using modular molds and attachments. We propose that the 3D-FAMM scheme can be broadly adopted to advance designs and tools in microfluidics.

## Materials and methods

### Fabrication process of the 3D-FAMM chips

Fig. 1b shows the fabrication process of the 3D-FAMM chip. The structures of the molds and the attachments were designed by computer-aided design software (Autodesk AutoCAD). Then, the molds and the attachments were 3D printed by a projection microstereolithography 3D printer (nanoArch® S130 and P140, BMF Precision Technology Co., China). A biocompatible resin (BIO, BMF Precision Technology Co., China) was used as the printing material. The frames and the trellis were fabricated by a computer numerical control (CNC) milling machine with a resolution of 25 µm (Runxing 1050 T, WeNext Technology Co., Ltd., China). The molds were assembled in the frame based on the microfluidic structure. Two silicone rubber bars were inserted into the interspace of the frame and molds. Stainless steel tubes were inserted into the reserved holes of molds, e.g., the inlets, the outlets and those used for interlayer connection. Methyl phenyl silicone oil (Andisil, AB Specialty Silicones, USA) dyed with crystal violet (Aladdin, China) was then dripped into the holes on the four corners of the frame. To fabricate a double-layer 3D-FAMM chip, the trellis was placed on the frame. The molds for the second layer were mounted into the trellis. PDMS (Sylgard 184, Dow Corning, USA) with a mixture ratio of 10:1 (weight ratio between prepolymer and cross-linker) was poured into the assembled molds and then cured at 60 °C. After 4 h, the PDMS microfluidic chip was demolded from the molds and washed in ethanol to remove residual silicone oil. Plasma treatment was applied to modify both sides of the PDMS microfluidic chip. The underside of the chip was sealed to a glass substrate, while the microstructures on the topside were covered by PDMS thin films with a size of 4 mm × 4 mm × 200 µm. Finally, the chip was heated for 2 h at 80 °C. The functional modules, such as the valve module, illumination module and camera module, were mounted into reserved vacancies. The thicknesses of the single-layer and double-layer 3D-FAMM chips were ~3 mm and 8 mm, respectively.

### Characterization of the molds and silicone rubber bars

The dimensions of the molds were measured using a digital micrometer caliper (MDH-25 M, Mitutoyo, Japan) with a resolution of 500 nm. Eighty molds were measured whose dimensions were consistent with the designed values. The gap filling process for the two assembled molds (Fig. 2b) was monitored by an optical contact angle meter (SL200B, KINO Scientific Instrument Inc., USA). The stress‒strain curves (Supplementary Fig. 3b, c) were measured by a stress gauge (PTR-1101, RHESCA, Japan) to quantitatively analyze the yield point and Young’s modulus. The Young’s moduli of the molds and the silicone rubber bars were 769 MPa and 7.8 MPa, respectively.

### Preparation of chemical solutions

In the concentration gradient generation, 150 mg of erioglaucine disodium salt (no. 861146, Sigma‒Aldrich, USA) was dissolved in 5 mL of deionized water to prepare a blue solution. The solution was filtered to remove impurities and undissolved particulates. The prepared blue solution and deionized water were used in the experiment.

In droplet generation and manipulation, mineral oil (no. M-3516, Sigma‒Aldrich, USA) was treated with surfactants (2.25% Span 80 (no. S6760, Sigma‒Aldrich, USA), 0.20% Tween 80 (no. S-8074, Sigma‒Aldrich, USA) and 0.02% Triton X-100 (no. T9284, Sigma‒Aldrich, USA)) to prepare the oil phase. The blue solution was used in the concentration gradient generation, and deionized water was used as the aqueous phase. The dynamic viscosity, *µ*, of the oil phase is 16 mPa·s. The chemicals were freshly prepared for each experiment.

### Simulation of fluid dynamics

The simulation of the fluid dynamics was performed using COMSOL Multiphysics® software (COMSOL Inc.). The 3D models of the microstructures were used in the simulation. In the simulation of droplet generation and manipulation, the pressure field, velocity field and flow trajectory of the fluid were conducted in a single-phase laminar flow system under a stationary state. In addition, a two-phase flow system with a level-set method was employed to model the process of droplet formation under a transient state. In the simulation of cell trapping, fluid flow behavior was calculated in a single-phase laminar flow system under a stationary state. Beads with a diameter of 24 μm were arbitrarily placed in the traps to imitate the existence of the cells.

### Cell culture and preparation

The HBEC3-KT human bronchial epithelial cell line (ATCC, Manassas, VA, USA) was cultured in Dulbecco’s modified Eagle’s medium (DMEM) with 10% fetal bovine serum (FBS), 100 U mL^−1^ penicillin and 100 μg mL^−1^ streptomycin. K562 and K562/ADR human myelogenous leukemia cell lines (Zhejiang Meisen Cell Technology Co., Ltd, China) were cultured in Roswell Park Memorial Institute (RPMI) 1640 culture medium supplemented with 10% fetal bovine serum (FBS), 100 U mL^−1^ penicillin and 100 μg mL^−1^ streptomycin. Then, 500 ng mL^−1^ Adriamycin (ADR, or doxorubicin) was added to the medium for the K562/ADR cell line to maintain drug resistance. All cells were cultivated in an incubator at 37 °C with 5% CO_2_.

### Supplementary information

Supplementary information

Supplemental Material1

Supplemental Material2

Supplemental Material3
