# Supplementary material for: 3D free-assembly modular microfluidics inspired by movable type printing
Source: Microsyst Nanoeng. 2023 Sep 11;9:111. doi: 10.1038/s41378-023-00585-1 (PMC10495351; doi:10.1038/s41378-023-00585-1)
Supplement: Supplementary file 1 — Supplementary information [file 41378_2023_585_MOESM1_ESM.docx]

Supplementary Information

**3D free-assembly modular microfluidics inspired by movable type printing**

Shaoqi Huang^1,†^, Jiandong Wu^2,†^, Lulu Zheng^1,†^, Yan Long^1^, Junyi Chen^1^, Jianlang Li^1^, Bo Dai^1^*, Francis Lin^3^*, Songlin Zhuang^1^, and Dawei Zhang^1^*

^1^ Engineering Research Center of Optical Instrument and System, the Ministry of Education, Shanghai Key Laboratory of Modern Optical System, University of Shanghai for Science and Technology, Shanghai, 200093, China

^2^ Institute of Biomedical and Health Engineering, Shenzhen Institute of Advanced Technology, Chinese Academy of Sciences, Shenzhen 518055, China

^3^ Department of Physics and Astronomy, University of Manitoba, Winnipeg, MB, R3T 2N2, Canada

**^†^** These authors contributed equally: Shaoqi Huang, Jiandong Wu, Lulu Zheng

* Correspondence and requests for materials should be addressed to B.D. (email: daibo@usst.edu.cn) or F.L. (email: francis.lin@umanitoba.ca) or D.Z. (email: dwzhang@usst.edu.cn)

**Supplementary Figures**

**
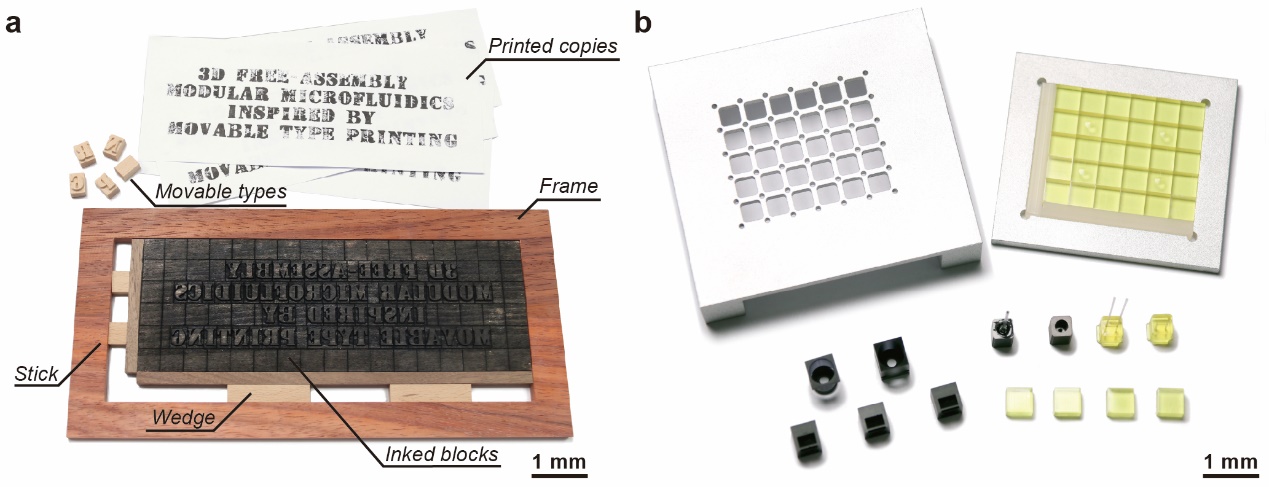
**

**Supplementary Fig. 1. Photograph of the components. a,** Photograph of the components in the movable type printing and the printed copies. **b,** Photograph of the components in the 3D-FAMM.


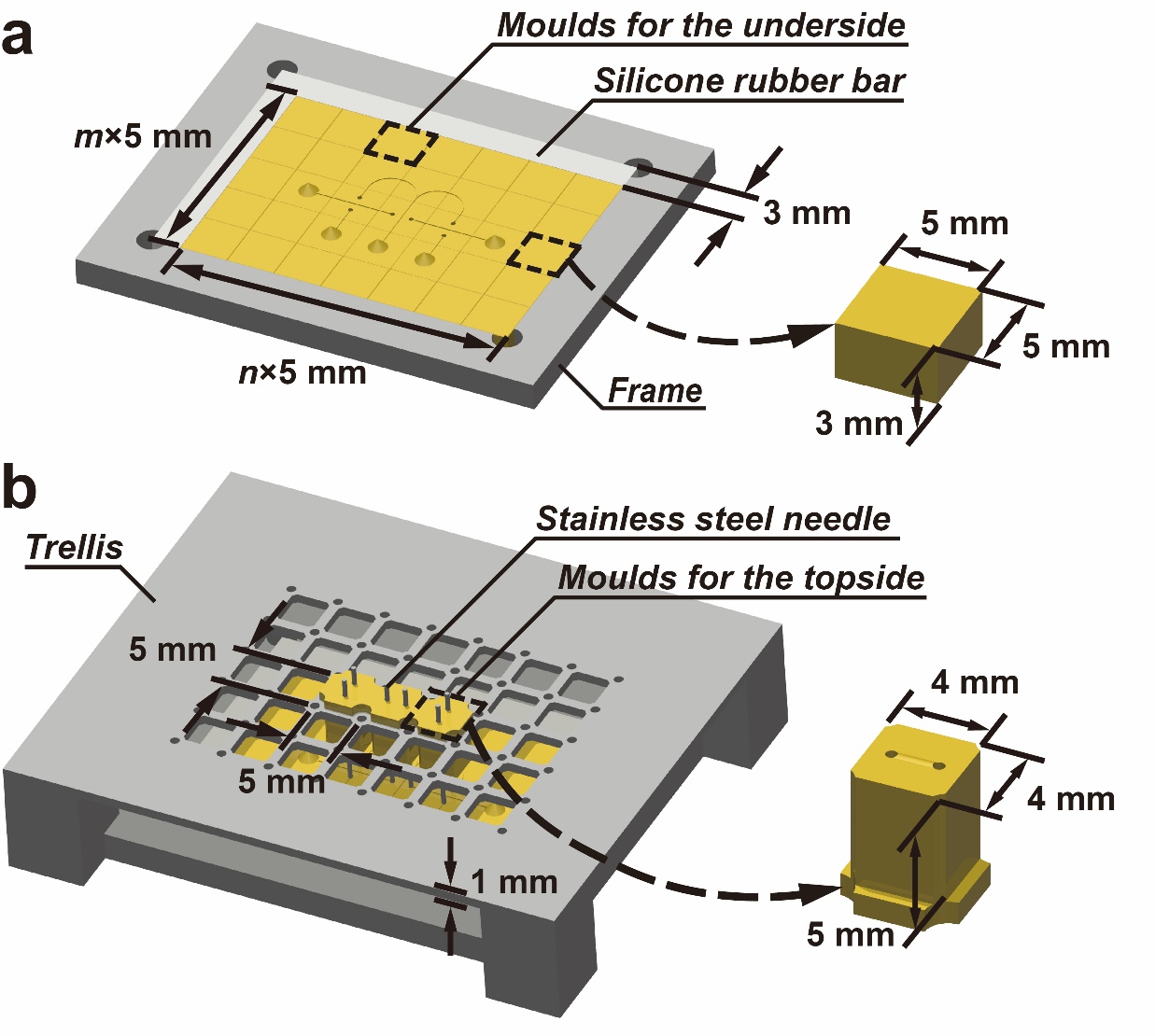


**Supplementary Fig. 2. Assembly of the molds for a double-layer microfluidic chip. a,** The molds for the first layer and the silicone rubber bars are assembled in the frame. **b,** The molds for the second layer are mounted into the trellis.


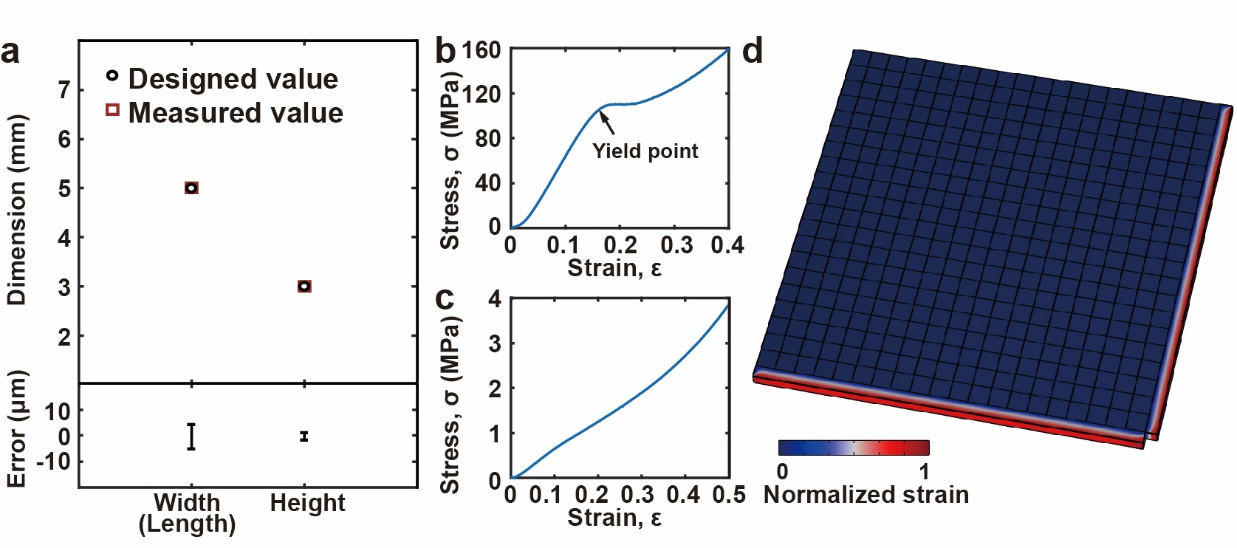


**Supplementary Fig. 3. Evaluation of the 3D-FAMM chips. a,** Measurement of the dimensions of the molds. **b,** The stress-strain behaviour in compression of the mold. **c,** The stress-strain behaviour in compression of the silicone rubber bar. **d,** Strain distribution of the assembly of the molds and the silicone rubber bars.


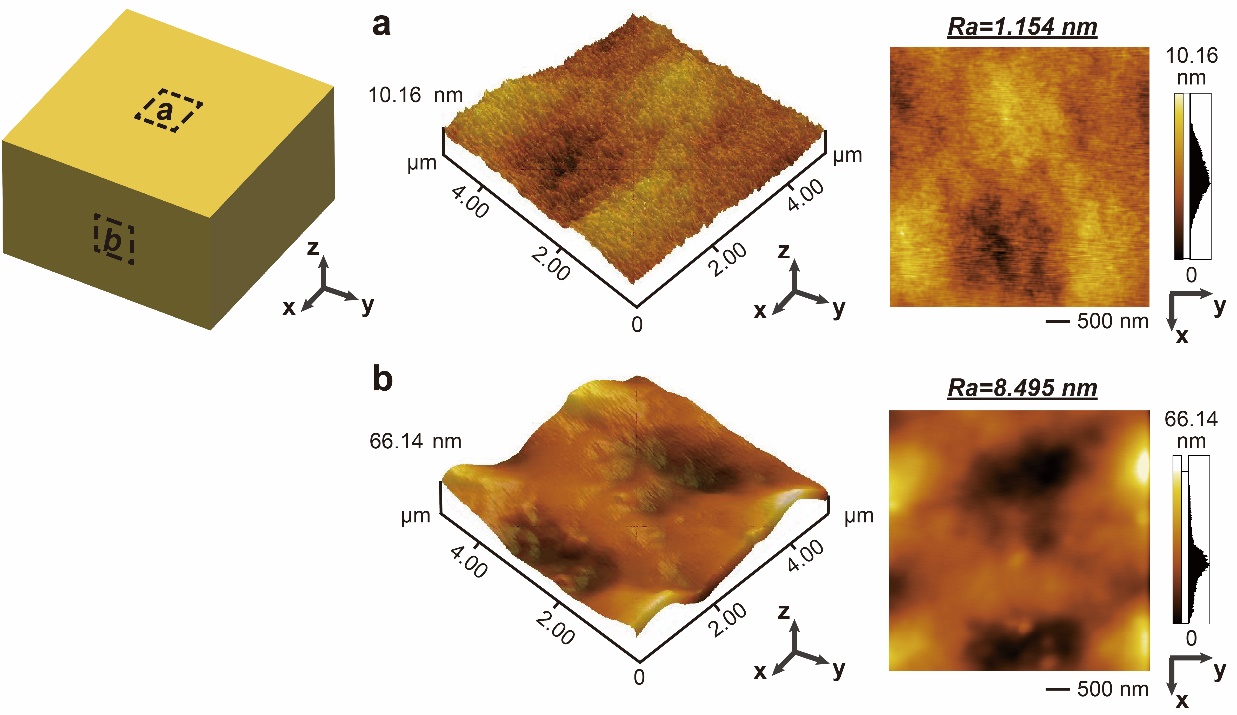


**Supplementary Fig. 4. Measurement of the surface roughness of the mold. a,** Surface roughness of the top of the mold. Ra<1.2 nm. **b,** Surface roughness of the side of the mold. Ra<8.5 nm.


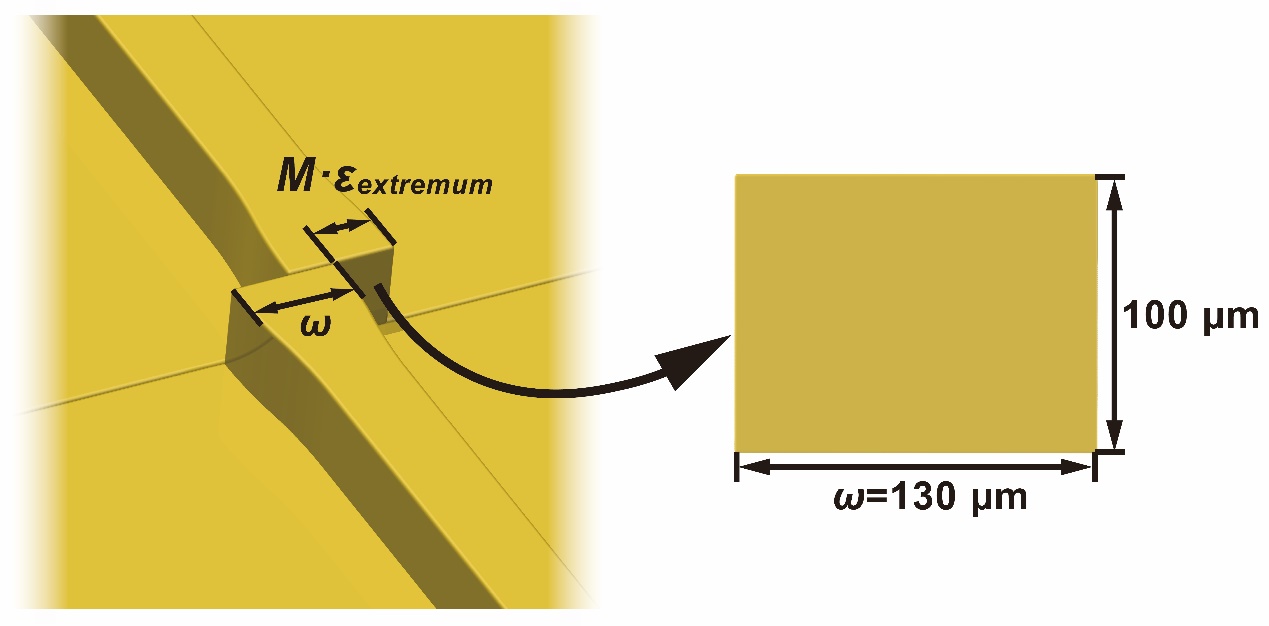


**Supplementary Fig. 5. The microchannel interface of the mold.** The microchannel interface is designed as 100 μm × 130 μm (height × width) to tolerate misalignment. The width of the microchannel interface, *ω*, should be larger than *Mε_extremum_*, where *M* is the number of the molds in series and *ε_extremum_* is the absolute extremum of the 3D printing in width. The measured absolute extremum of the error, *ε_extremum_*, is less than 5 μm.


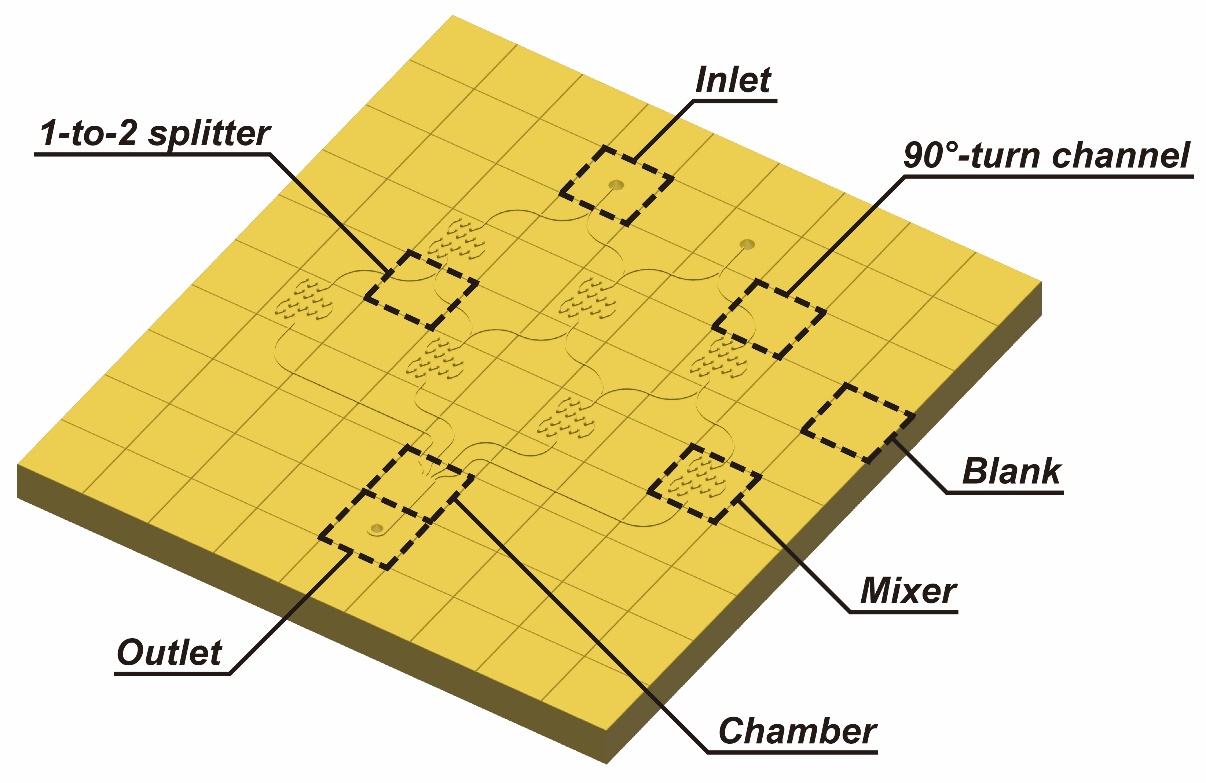


**Supplementary Fig. 6. Schematic diagram of the assembled Christmas-tree-shaped microfluidic concentration gradient generator.**


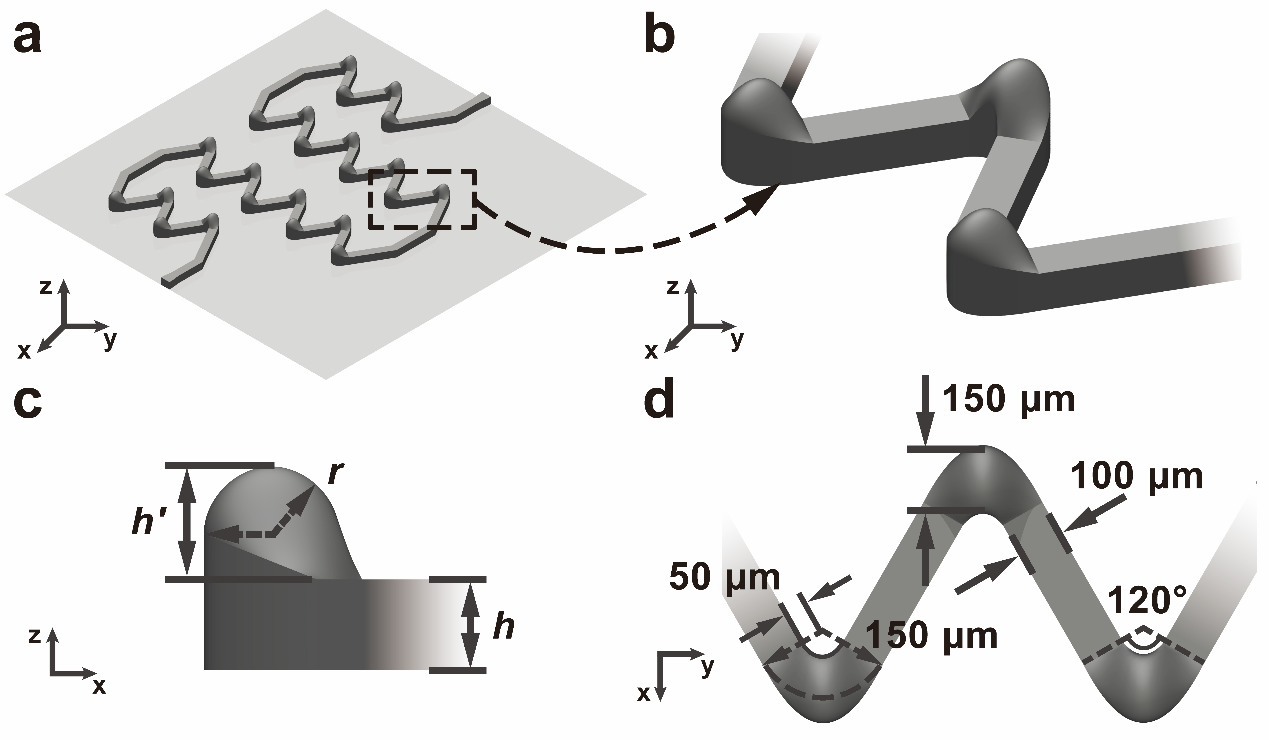


**Supplementary Fig. 7. Structural design of the 3D mixer mold. a,** The mold of the 3D mixer. **b,** The 3D mixer mold has a zigzag structure. There is a slight bulge at every turn. **c,** Side view of the 3D mixer. **d,** Top view of the 3D mixer. *h'=1.25h* and *r=0.6h'*, where *h* is the height of main channel, *h'* is the height of the bulge, and *r* is the radius of the bulge.


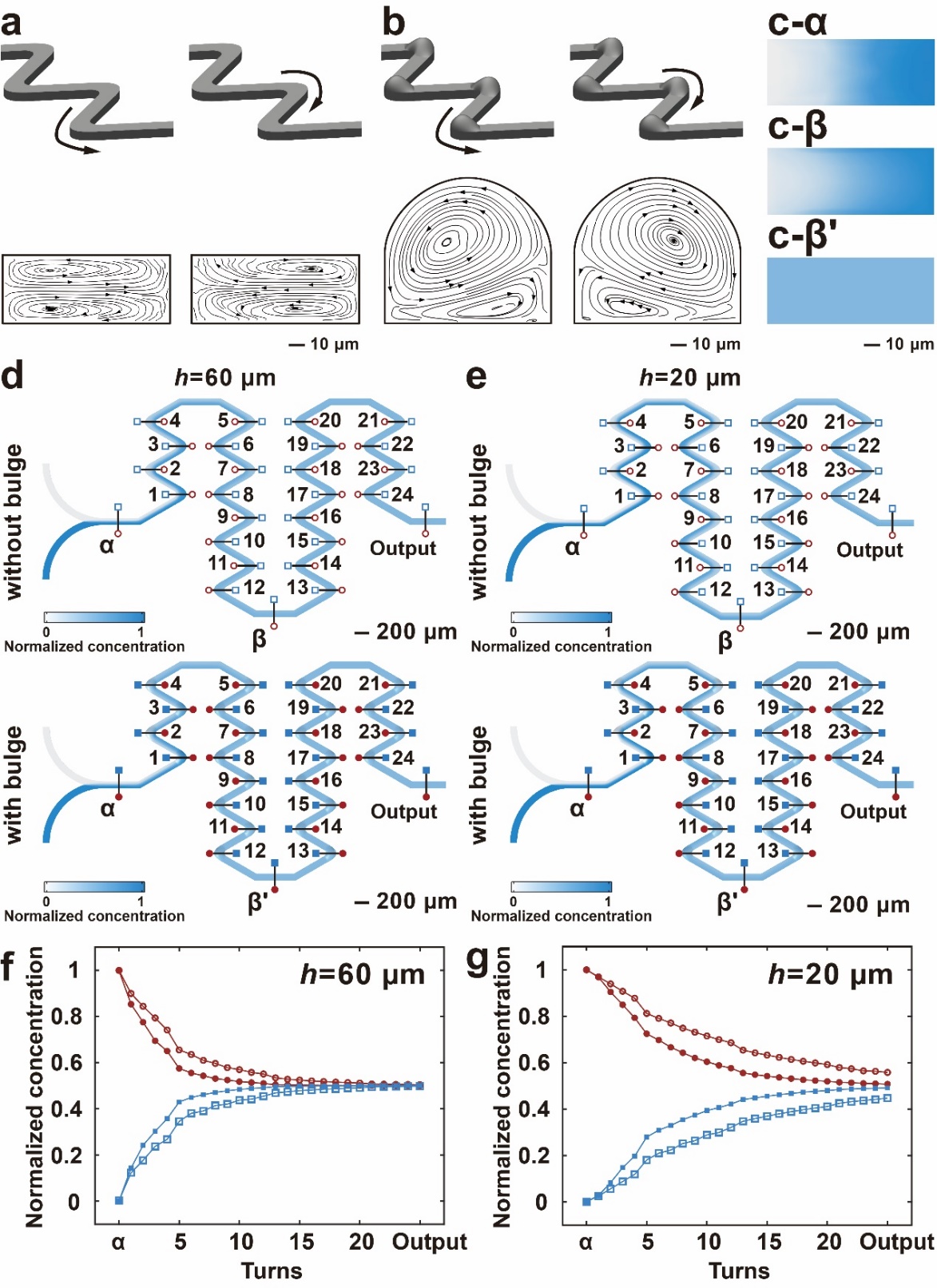


**Supplementary Fig. 8. Calculation of the mixing efficiency of the mixers with and without bulge at the turns. a and b,** The flow trajectories at the turn in the conventional zigzag-shaped mixer and the 3D mixer with the bulges at the turns. **c,** Concentration distribution on the cross section of the microchannel at points α, β and β', as marked in **d** and **e**. **d and e,** Concentration distribution of the mixers along the flow direction. **f and g,** The plots of the normalized concentration. Hollow markers: the conventional mixer. Filled markers: the 3D mixer. Blue square markers: the concentration on the left side of the microchannel. Red circle markers: the concentration on the right of the microchannel.


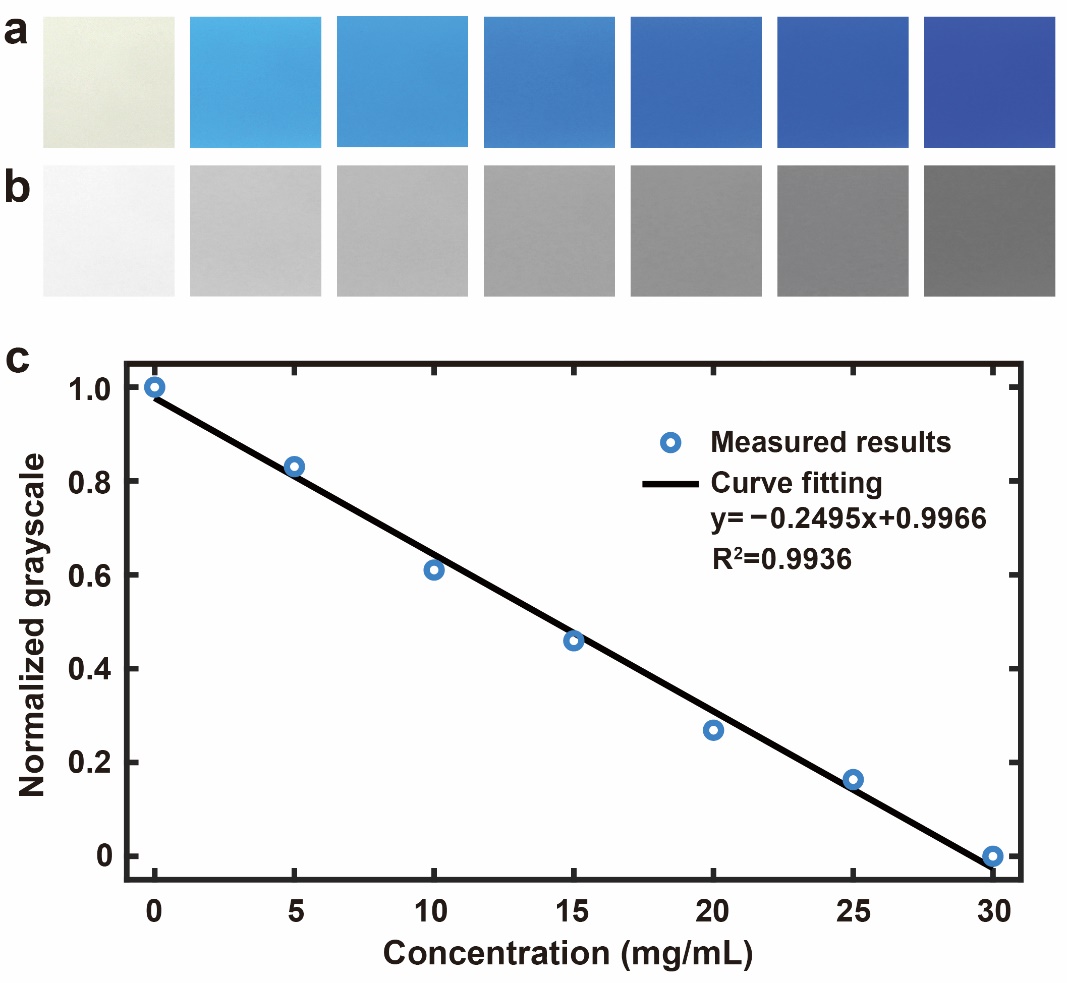


**Supplementary Fig. 9. The relation between the colour and the solution concentration of the dye. a,** The images of the blue dyed solution. **b,** The grayscale images converted from the colour images. **c,** The relation between the concentration of the dye and the grayscale values. R^2^ is the goodness of fit.


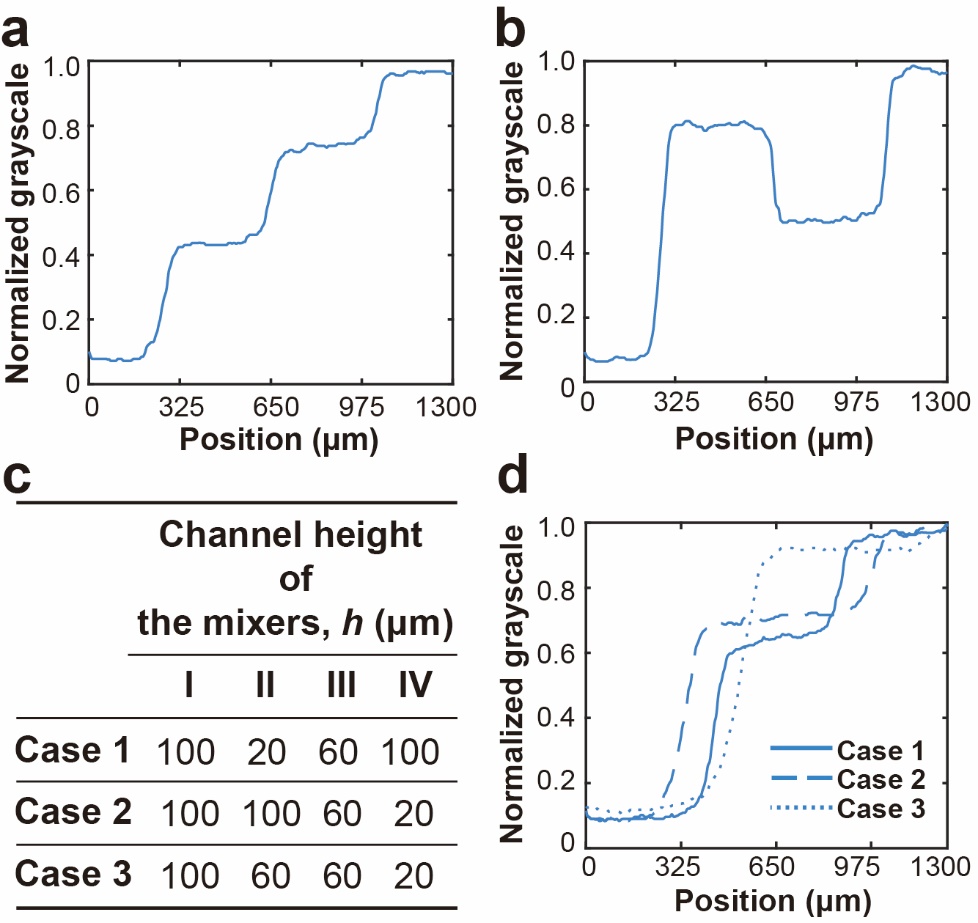


**Supplementary Fig. 10. Concentration gradient profiles. a,** Concentration gradient based on the concentration profile along the dashed line in **Fig. 4b**. **b,** Concentration gradient based on the concentration profile along the dashed line in **Fig. 4d**. **c,** A list of the channel height of the mixers. **d,** Concentration gradient based on the concentration profile along the dashed lines in **Fig. 4f-h**.


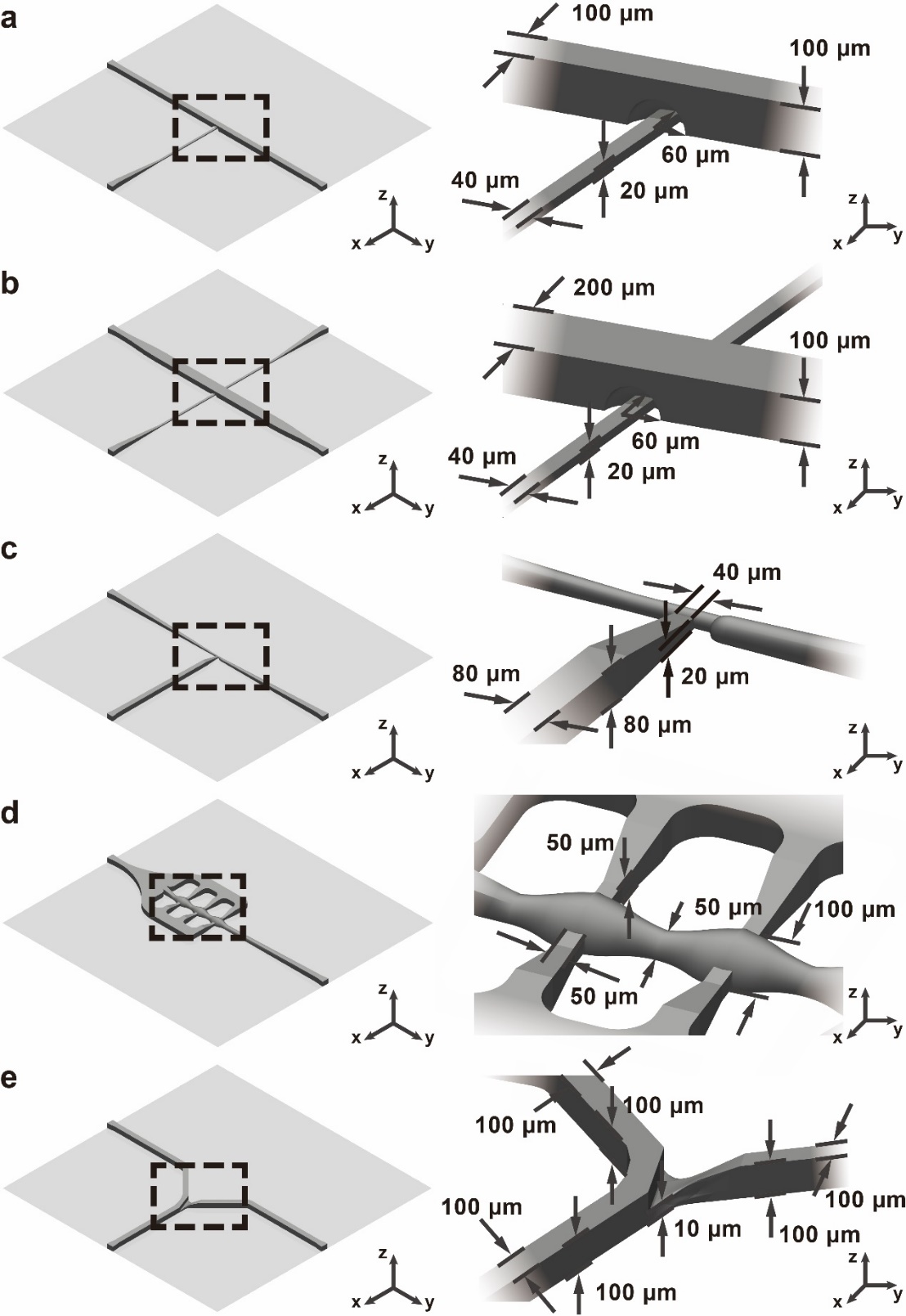


**Supplementary Fig. 11. Structures of the molds used in the droplet generation and manipulation. a and b,** The molds for the droplet generation. The droplet generator has a 3D T-junction with a nozzle configuration. **c,** The mold for the droplet injection. The main channel narrows before the injection. **d,** The mold for the droplet merging. The main channel has three chambers in series and two auxiliary channels are on the two sides connecting to the chambers. **e,** The mold for the droplet splitting. The droplet splitter has a Y-shaped structure. The height of one of the branches is lowered to 10 µm, forming an obstacle to intercept particles.


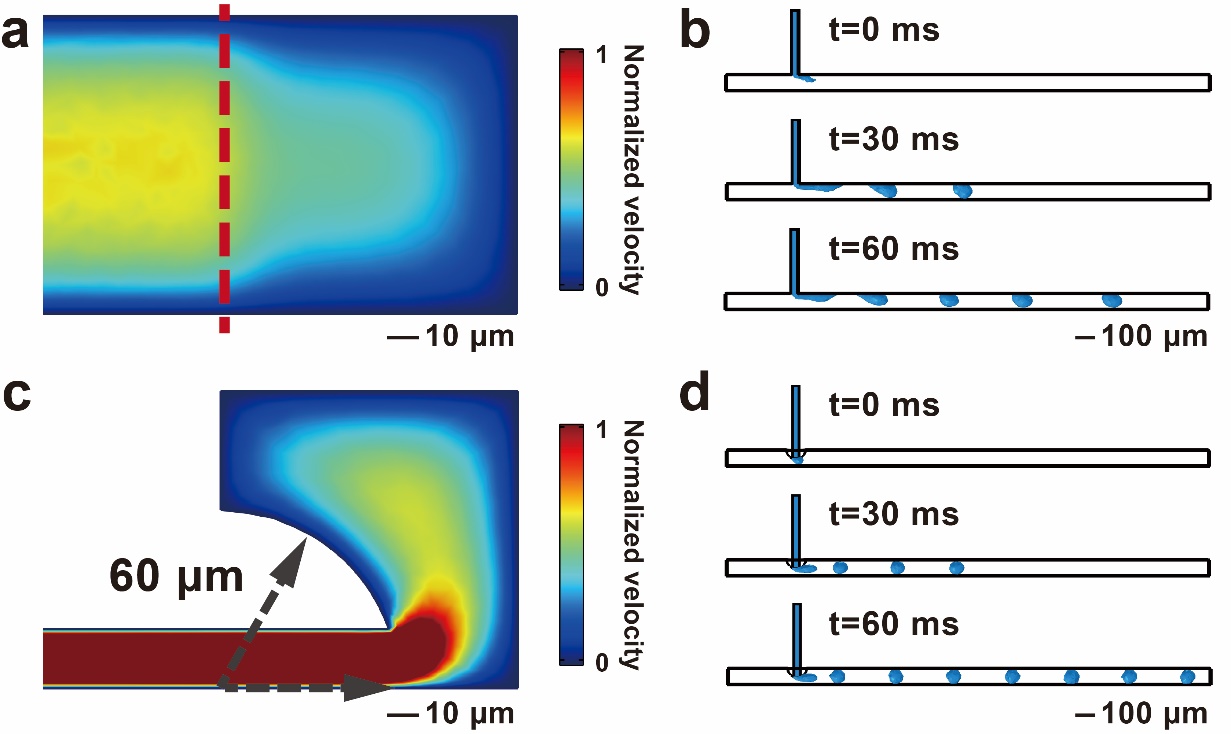


**Supplementary Fig. 12. Simulation of the droplet generation at T-junction. a and c,** Normalized velocity field on the cross section at T-junction. **b and d,** The montages of the droplet generation. **a and b,** The conventional T-junction structure. **c and d**, The 3D T-junction with the nozzle configuration.


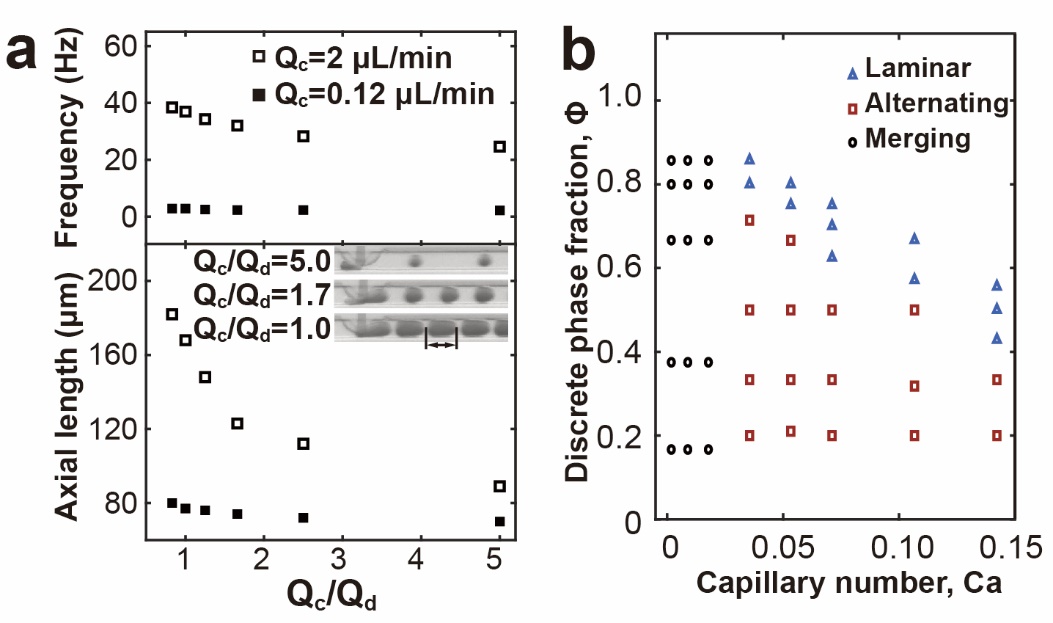


**Supplementary Fig. 13. Analysis of the droplet generation. a,** The influence of the flow rate ratio over the droplets generation. **b,** The phase diagram of the droplet generation. *Ca=µQ_c_/σS*, where *µ* is the dynamic viscosity of the continuous phase, *Q_c_* is the flow rate of the continuous phase, *σ* is the interfacial tension between the two fluid phases, and *S* is the area of the cross section of the channel. *Φ=(Q_d1_+Q_d2_)/(Q_c_+Q_d1_+Q_d2_)*, where *Q_d1_* and *Q_d2_* are the flow rate of the two discrete phases.


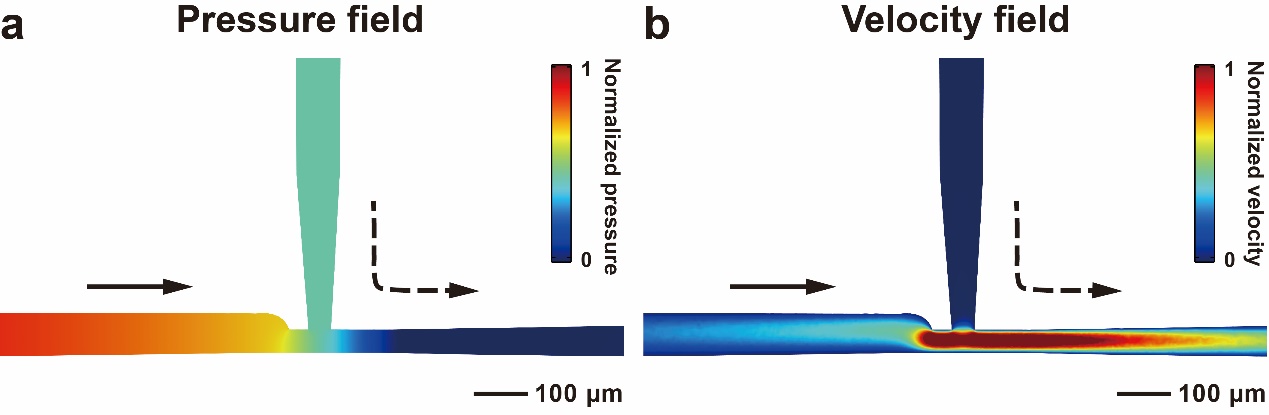


**Supplementary Fig. 14. Simulation of the passive droplet injection. a,** The normalized pressure field. The pressure suddenly drops at the junction, allowing the injection with a low pressure. **b,** The normalized velocity field.


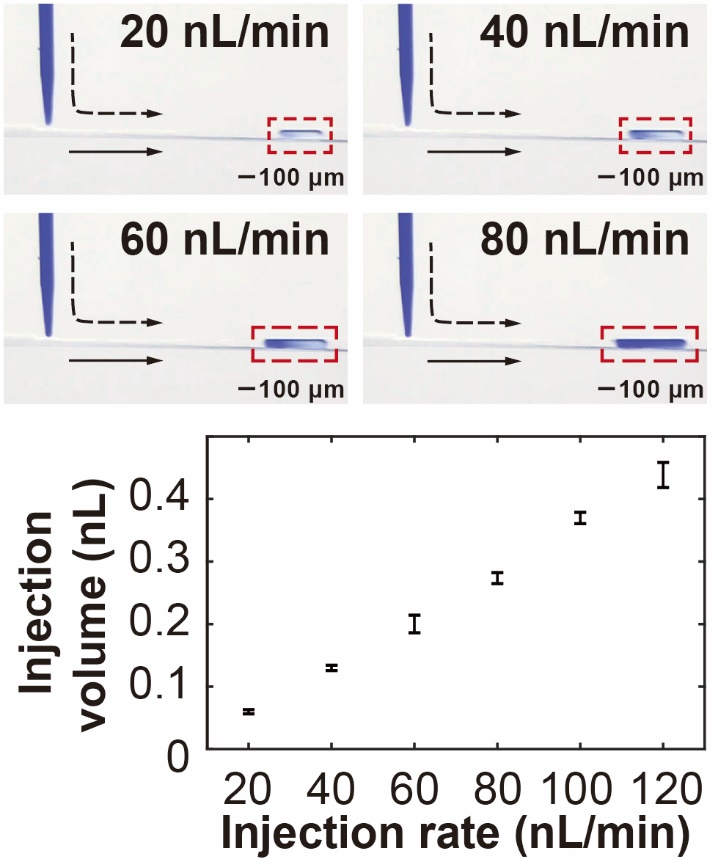


**Supplementary Fig. 15. The influence of the injection rate on the injection volume.**


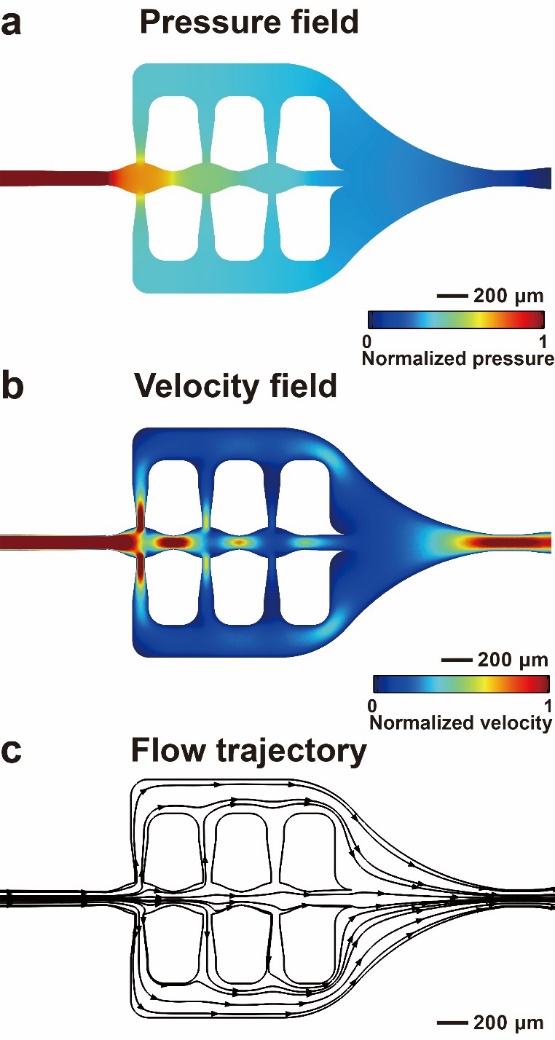


**Supplementary Fig. 16. Simulation of the passive droplet merging. a,** The normalized pressure field. **b,** The normalized velocity field. **c,** The flow trajectory.


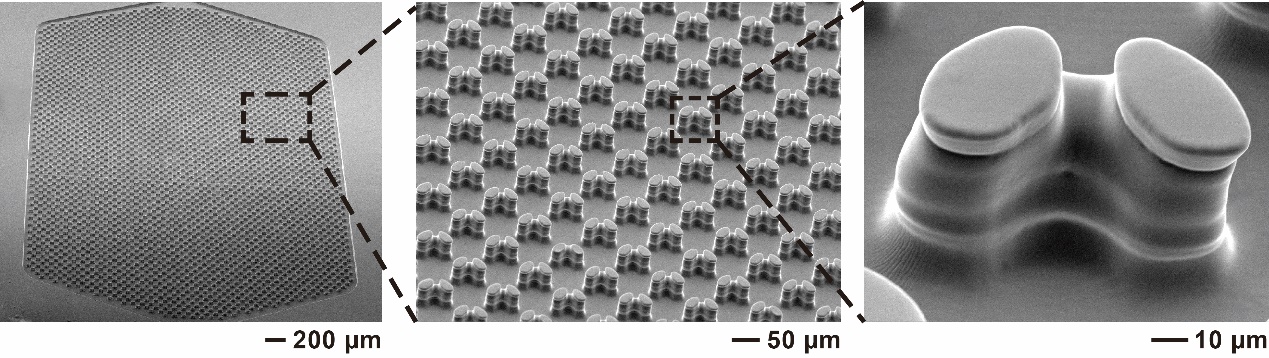


**Supplementary Fig. 17. Scanning electron microscopic (SEM) images of the cell trapping module.**


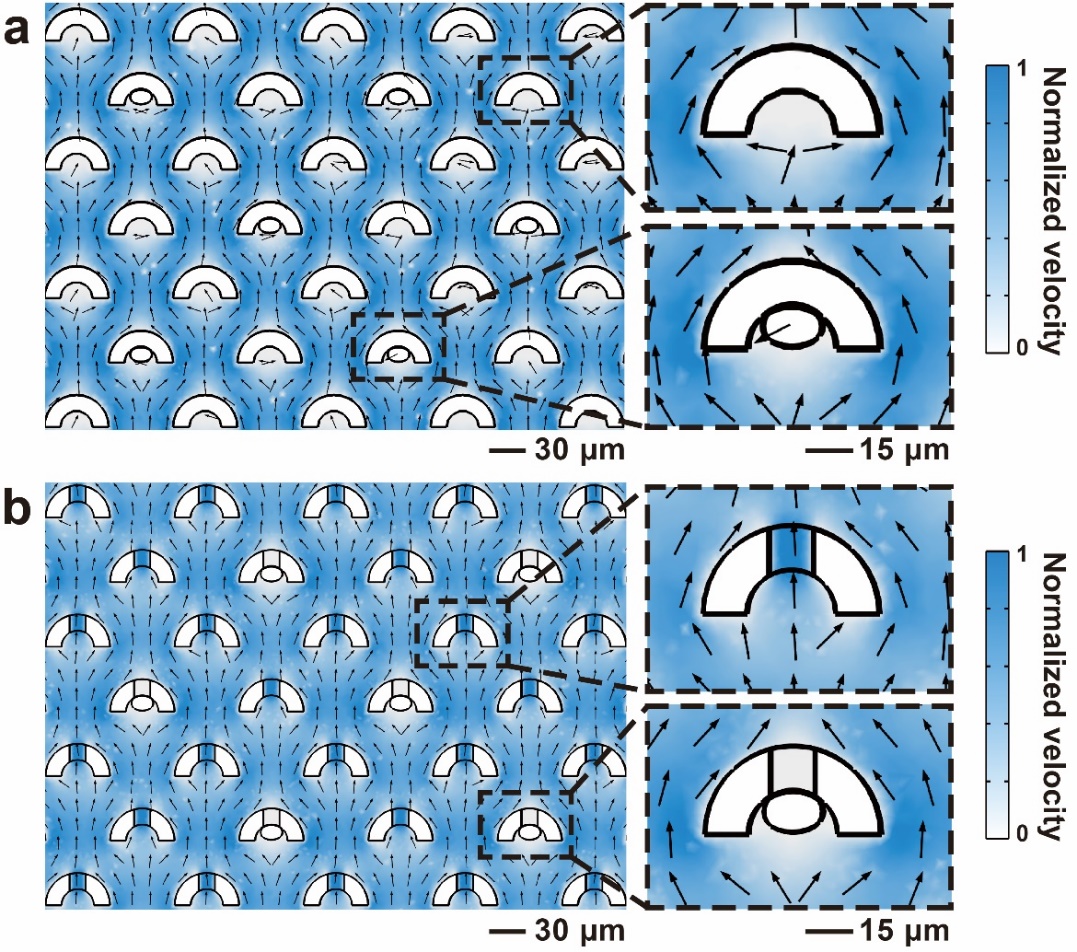


**Supplementary Fig. 18. Normalized velocity field of the trapping. a,** The traps have no gaps. **b,** The 3D traps have gaps on the bottom. The height of the gap is 5 μm. In the simulation, some particles are placed in a portion of traps.


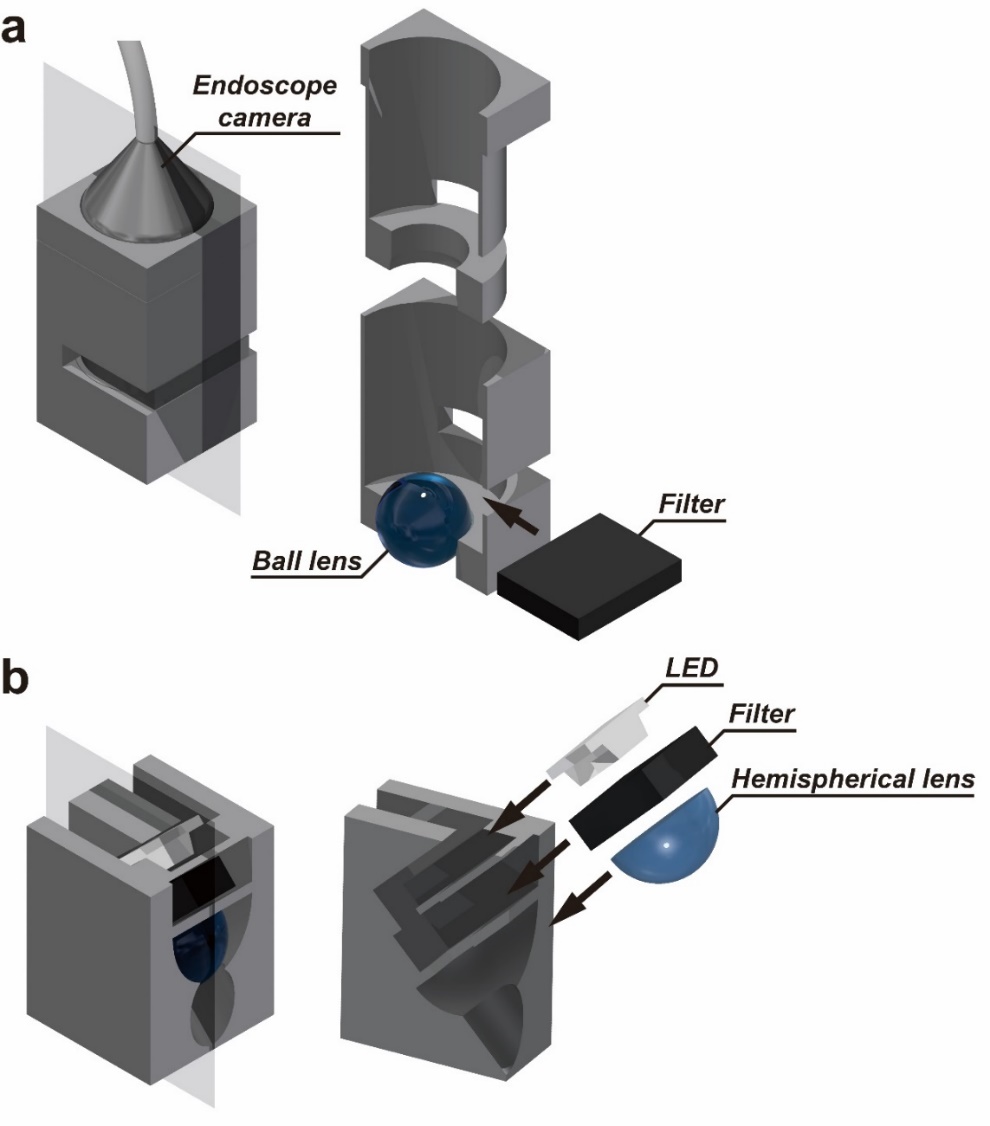


**Supplementary Fig. 19. Schematic diagram of the illumination module and the camera module**. **a,** The camera module consists of an endoscope camera, an optical bandpass filter (optional) and a ball lens. **b,** The illumination module consists of a LED, an optical bandpass filter (optional) and a hemispherical lens.


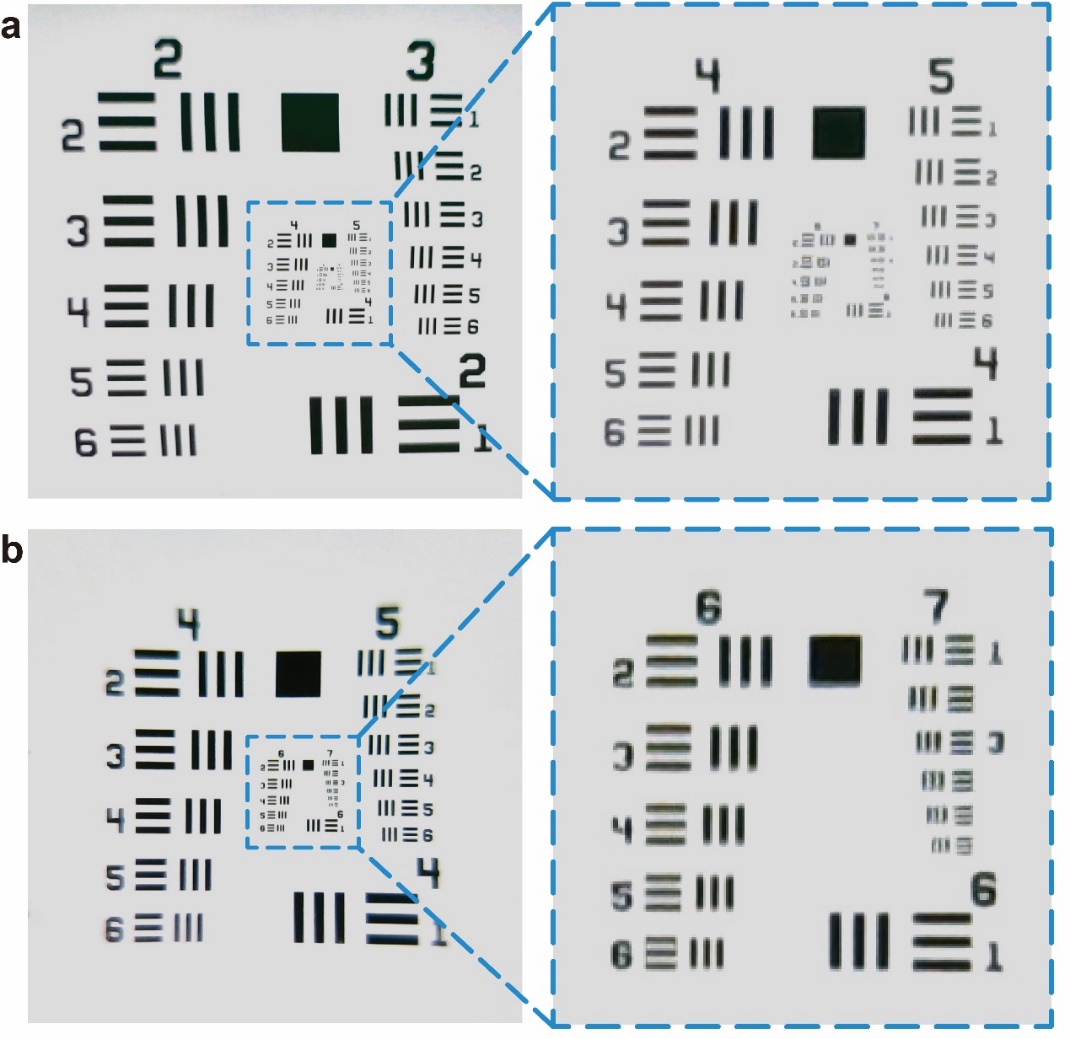


**Supplementary Fig. 20. Microscopic imaging by using the camera module. a,** The image captured by the camera module without using the lens. **b,** The image captured by the camera module with the 3 mm ball lens.

**Supplementary Tables**

**Supplementary Table 1.** The 3D-FAMM modules.

| **Mold for**  **the underside** | **Magnification** | **Description** |
| --- | --- | --- |
| 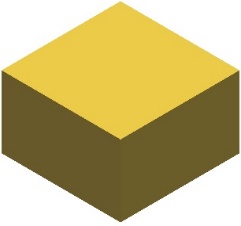 |  | Blank |
| 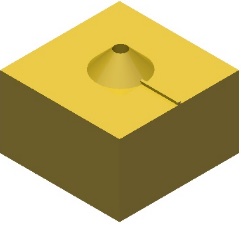 |  | Inlet/outlet |
| 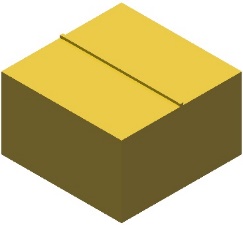 |  | Straight channel |
| 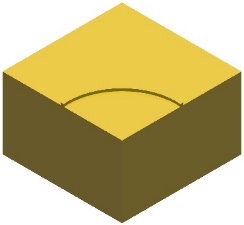 |  | 90°-turn channel |
| 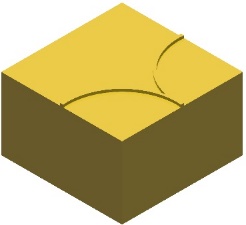 |  | 1-to-2 fluid splitter/combiner |
| 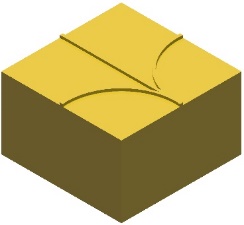 |  | 1-to-3 fluid splitter/combiner |
| 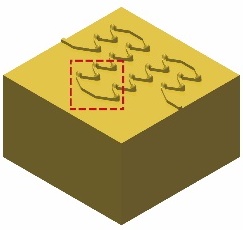 | 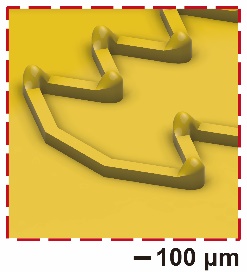 | Mixing |
| 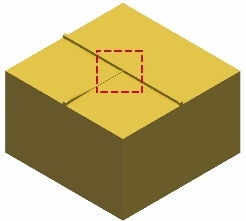 | 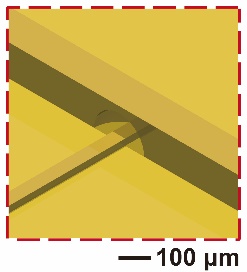 | Droplet generation  (single droplet) |
| 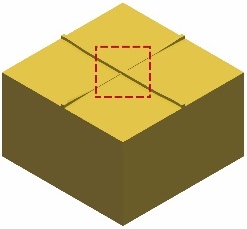 | 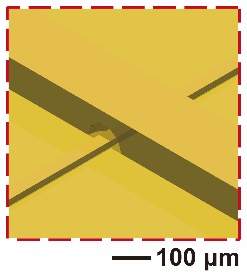 | Droplet generation  (alternating droplet, merging droplet and laminar flow) |
| 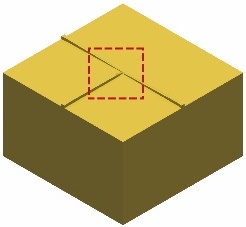 | 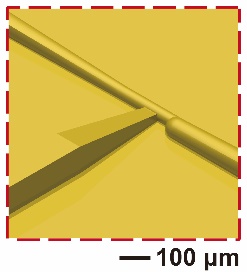 | Droplet injection |
| 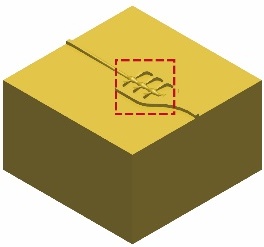 | 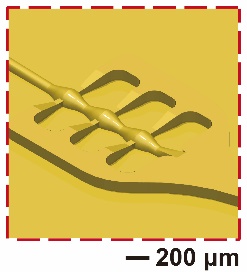 | Droplet merging |
| 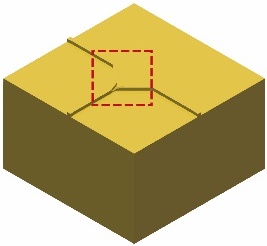 | 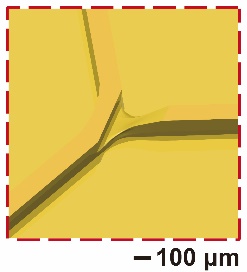 | Droplet splitting |
| 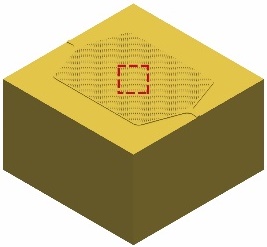 | 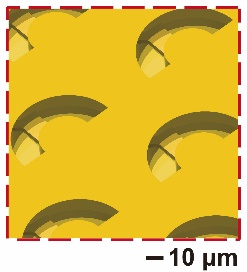 | Cell trapping |
| 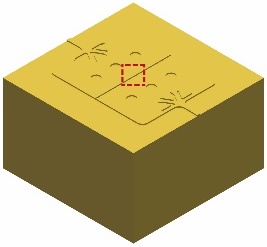 | 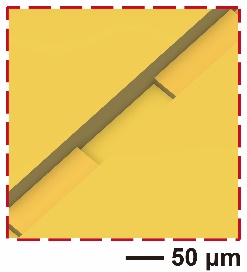 | Cell co-culture |
| 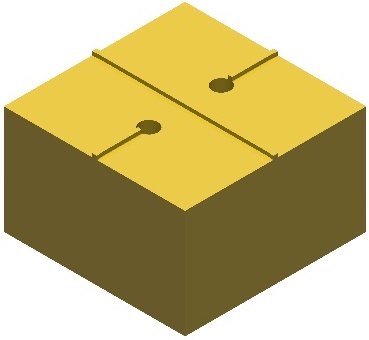 |  | Bypass microchannel  (underside) |
|  | | |
| **Mold for**  **the topside** | **Magnification** | **Description** |
| 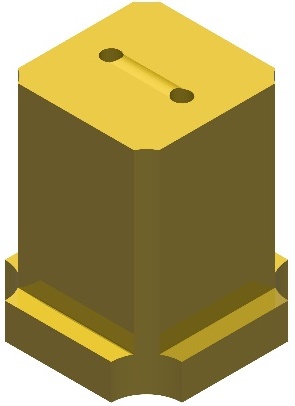 |  | Bypass microchannel  (topside) |
| 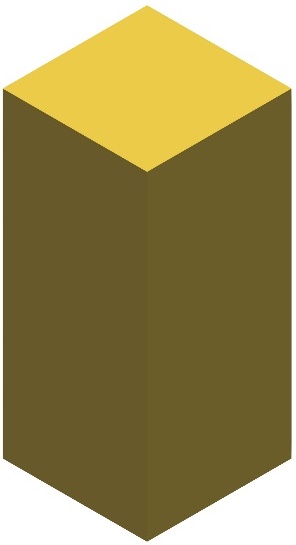 |  | Placeholder |
|  | | |
| **Attachment** | **Sectional view** | **Description** |
| 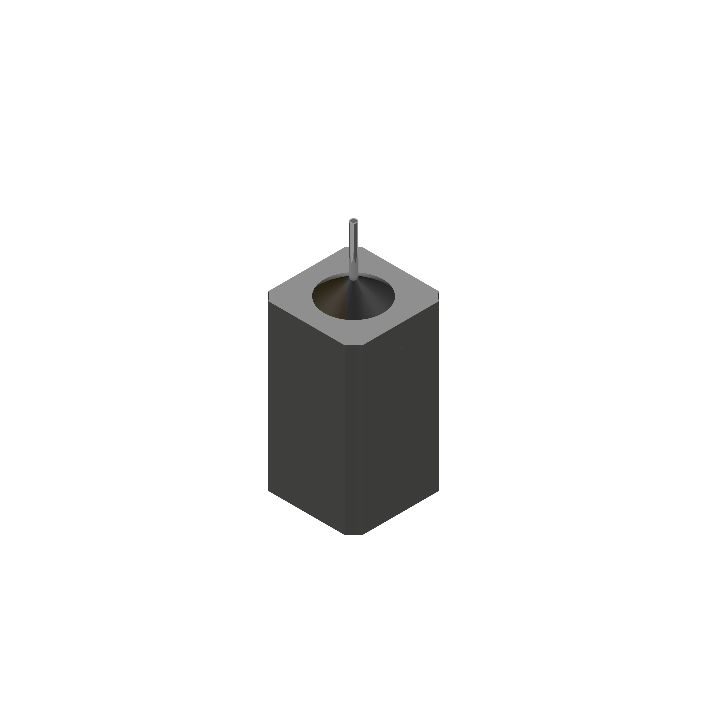 | 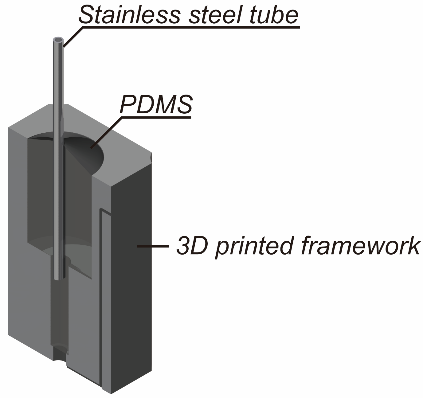 | Pneumatic valve |
| 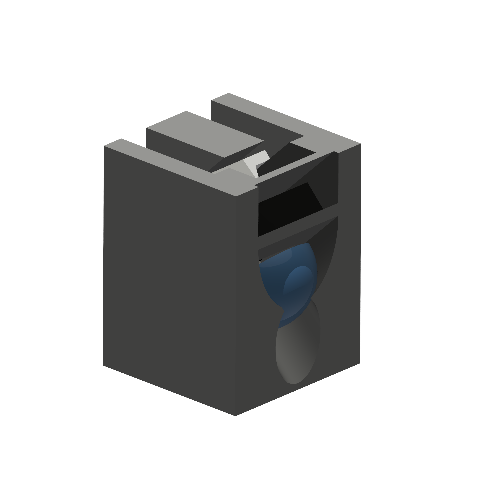 | 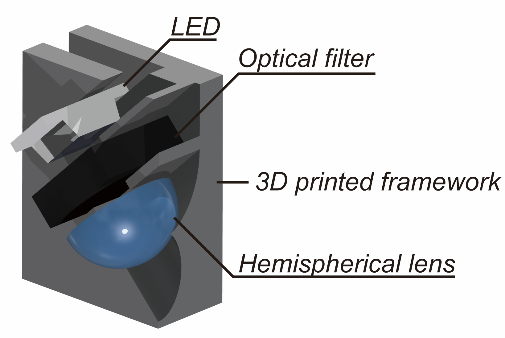 | Illumination |
| 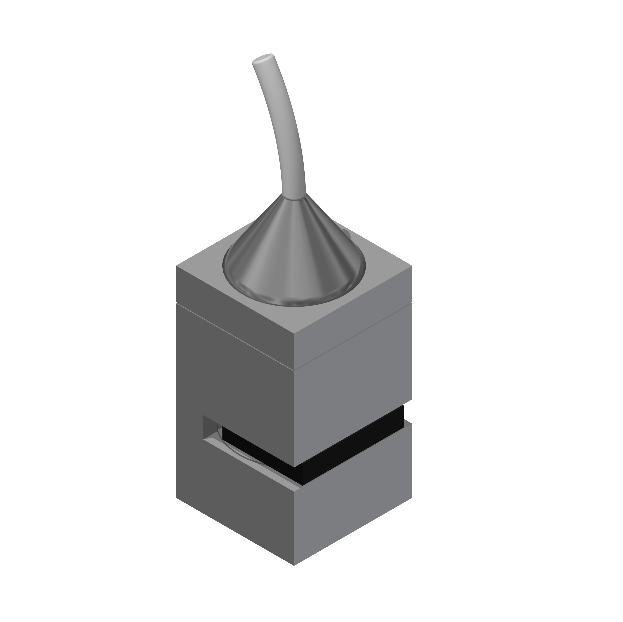 | 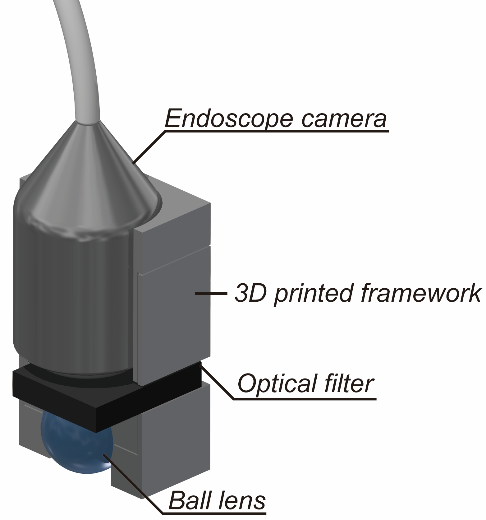 | Microscopic camera |

**Supplementary Movie**

***Movie S1***

Fabrication process of the single-layer microfluidic chip with the S-shaped microchannel.

***Movie S2***

Fabrication process of the double-layer microfluidic chip with the m-shaped microchannel equipped with the valve.

***Movie S3***

Demo 1: Droplet generation – single droplets

Demo 2: Droplet generation – merging droplets

Demo 3: Laminar flow

Demo 4: Droplet injection

Demo 5: Droplet merging

Demo 6: Droplet splitting – symmetric Y-junction structure

Demo 7: Droplet splitting – asymmetric Y-junction structure

**Supplementary Notes**

***Supplementary Note 1: Investigation of the mold deformation***

The stress-strain behaviours of the molds and the silicone rubber bars were measured (Supplementary Fig. 3b, c). Besides, the deformation of the molds was analysed using COMSOL Multiphysics® software. The solid mechanics model was adopted with a stationary state to investigate the strain of mold and silicone rubber bar. Supplementary Fig. 3d illustrates the strain distribution of the mold assembly in which 20×20 molds with the size of the worst positive error (5.01 mm) and the two silicone rubber bars are compressed into a 102.8×102.8 mm^2^ square. The silicone rubber bars dissipate most of the strain energy and the strain applied on the molds is very low.

***Supplementary Note 2: Colorimetric analysis of the concentration***

A colorimetric method was employed to map the colour of the solution to the concentration of the dye. The blue solution with different concentration was prepared by dissolving erioglaucine disodium salt (no. 861146, Sigma-Aldrich, USA) ranging from 10 mg to 60 mg in the 2 mL deionized water. Then, the blue solution was injected into the 3D-FAMM chip that has microchambers with the height of 100 µm. Images of the blue solution were recorded (Supplementary Fig. 8a). 100×100-pixel matrices were extracted from the images. After converting the colour images to grayscale images (Supplementary Fig. 8b), the mean values, *I_Mean_*, of the pixel matrices were calculated. The grayscale value obtained from the images of the solution with the highest concentration solution, *I_Max_*, and the deionized water, *I_Base_*, were used as the references. The grayscale values for the solution with other concentration was normalized as follows

$$I_{Norm}=\frac{I_{Mean}-I_{Base}}{I_{Max}-I_{Base}}$$

The normalized grayscale values of the blue solution with different concentration and the deionized water were plotted on Supplementary Fig. 8c. Linear regression was applied to find the relation between the concentration and the normalized grayscale. The high goodness of fit, R^2^, indicated that the concentration of the blue solution could be represented by the normalized grayscale.

***Supplementary Note 3: Droplet generation using 3D-FAMM***

In the T-junction droplet generator, the influx of the discrete phase from the nozzle gradually enlarges and impedes the continuous phase in the main channel, resulting in the increase of the pressure of the continuous phase before the T-junction. Then, the continuous phase starts to squeeze the tongue of the discrete phase. Finally, the neck connecting the tongue with the discrete phase inside the nozzle breaks and a droplet is generated.

In the droplet generator with two T-junction structures, when the flow rate of the continuous phase is low, *i.e.* under the condition of low *Ca*, the two discrete phases merge at the T-junction because the pressure of the continuous phase is insufficient to break the individual discrete phases. Then, the merged discrete phase blocks the main channel and the continuous phase with the enhanced pressure to break the merged phase into droplets. If the flow rate of the continuous phase is relatively large and comparable with the sum of the flow rates of the discrete phases, the continuous phase can apply adequate pressure on the two discrete phases respectively to break them into the alternating droplets. Otherwise, the laminar flow is formed where the continuous phase is incapable of breaking the discrete phases into droplets but can separate them on the two sides of the channel.
